# Biophysical and functional study of CRL5^Ozz^, a muscle specific ubiquitin ligase complex

**DOI:** 10.1038/s41598-022-10955-w

**Published:** 2022-05-12

**Authors:** Yvan Campos, Amanda Nourse, Ajay Tanwar, Ravi Kalathur, Erik Bonten, Alessandra d’Azzo

**Affiliations:** 1 Department of Genetics, St. Jude Children’s Research Hospital, 262 Danny Thomas Place, Memphis, TN 38105 USA; 2 Department of Structural Biology, St. Jude Children’s Research Hospital, 262 Danny Thomas Place, Memphis, TN 38105 USA; 3 Department of Chemical Biology and Therapeutic, St. Jude Children’s Research Hospital, 262 Danny Thomas Place, Memphis, TN 38105 USA

**Keywords:** Cell biology, Molecular biology, Structural biology

## Abstract

Ozz, a member of the SOCS-box family of proteins, is the substrate-binding component of CRL5^Ozz^, a muscle-specific Cullin-RING ubiquitin ligase complex composed of Elongin B/C, Cullin 5 and Rbx1. CRL5^Ozz^ targets for proteasomal degradation selected pools of substrates, including sarcolemma-associated β-catenin, sarcomeric MyHC_emb_ and Alix/PDCD6IP, which all interact with the actin cytoskeleton. Ubiquitination and degradation of these substrates are required for the remodeling of the contractile sarcomeric apparatus. However, how CRL5^Ozz^ assembles into an active E3 complex and interacts with its substrates remain unexplored. Here, we applied a baculovirus-based expression system to produce large quantities of two subcomplexes, Ozz–EloBC and Cul5–Rbx1. We show that these subcomplexes mixed in a 1:1 ratio reconstitutes a five-components CRL5^Ozz^ monomer and dimer, but that the reconstituted complex interacts with its substrates only as monomer. The in vitro assembled CRL5^Ozz^ complex maintains the capacity to polyubiquitinate each of its substrates, indicating that the protein production method used in these studies is well-suited to generate large amounts of a functional CRL5^Ozz^. Our findings highlight a mode of assembly of the CRL5^Ozz^ that differs in presence or absence of its cognate substrates and grant further structural studies.

## Introduction

During development and maturation of the muscle fibers, a series of events occur that lead muscle progenitor cells through morphological and functional transitions. These events require the tight control of the abundance of both structural and regulatory proteins, as well as the selective degradation of embryonic and fetal isoforms that are replaced by their adult counterparts^1^. The ubiquitin proteasome system^2^ plays a central role in many of these processes. Protein ubiquitination is a post-translational modification that involves the covalent attachment of ubiquitin (Ub) or ubiquitin chain to the e-amino group of a lysine residue on a target substrate^3–5^. Depending on the type of ubiquitination, the Ub-marked substrate is either destined for proteasomal degradation or assumes a conformation that favors its recognition by other protein partners and its intracellular trafficking^6^. The ubiquitination reaction requires the sequential actions of an E1-activating enzyme, an E2-conjugating enzyme, and finally an E3 ubiquitin ligase^3–6^. The latter defines the selectivity of the target substrates as well as the sites where ubiquitination occurs^7^.

Ozz, a member of the suppressor of cytokine signaling (SOCS)-box family of proteins^8^, is the substrate-binding component of the Cullin-RING Ubiquitin Ligase (CRL), CRL5^Ozz^ (formerly referred to as Ozz-E3), consisting of Elongin B and Elongin C (EloBC), the Cullin protein, Cul5, and the RING-finger protein, Rbx1^1,9–12^**.** Within the Ozz primary structure, the SOCS-box domain is located at the C-terminus of the protein and embeds the EloBC binding site that directs the assembly of the rest of the complex. The substrate recognition site consists of two adjacent neuralized homology repeats (NHR) located at the N-terminus^13,14^. The NHR motif is present in the drosophila protein Neuralized (Neur), which is a single-chain E3 ligase involved in the degradation of Delta and the specification of neural cell fate^13–15^.

CRL5^Ozz^ is a unique member of the CRL family of E3 ligases. It is tissue specific, being expressed exclusively in striated muscle; it targets and ubiquitinates selected subpopulations of muscle proteins, which have the common attribute of being fully assembled and components of multiprotein complexes that are linked to the actin cytoskeleton. These include the plasma membrane-associated β-catenin^10^, the fully assembled, sarcomeric MyHC_emb_^1^ and a distinct pool of the Alix/PDCD6IP scaffold protein that bridges the subcortical actomyosin network with membrane complexes^9^. To form an active CRL, Ozz needs to complex with the other components, a process that adds an extra tier to the regulation of substrate recognition and ubiquitination by this ligase^10^. Proper function and regulation of CRL5^Ozz^ assure the assembly and stability of the contractile sarcomeric unit, as well as the interconnection between membrane complexes and the actin cytoskeleton in skeletal muscle. Ozz ablation in vivo results in defects in myofibrillogenesis and sarcomere assembly^1,9,10^. However, the full spectrum of CRL5^Ozz^ functions is still unfolding, and only a few of its substrates and their cellular roles in striated muscle have been investigated.

To begin to address these questions we have now investigated how CRL5^Ozz^ assembles into an active E3 complex and how it interacts with three of its substrates. To this end, we have developed a baculovirus (BV)-based expression system in insect cells to produce large quantities of the CRL5^Ozz^ complex and its individual substrates. We have successfully reconstituted a functional and active CRL5^Ozz^ by combining two, separately expressed subcomplexes, Ozz–EloBC and Cul5–Rbx1. Biophysical analyses of the in vitro reconstituted CRL5^Ozz^ alone or combined with individual substrates reveal a mode of assembly that differs in absence vs presence of the substrates. Our results hold promise for future structural studies of CRL5^Ozz^ in combination with its substrates.

## Materials and methods

### Generation of CRL5^Ozz^ BV

Full-length cDNA clones encoding human Ozz (Ozz), Elongin B, Elongin C, Cul5 and Rbx1 were amplified by RT-PCR (Table 1) from commercially available Human RNA (Clontech-Takara Bio). Ozz and Rbx1 fused His tag were cloned into the pFastBac HTb and pFastBac HTc respectably, while Elongin B, Elongin C and Cul5 were cloned into pFastBac1 vector (Life Technologies). The generated plasmids were transformed into DH10Bac competent cell to generate the individual Ozz E3 components bacmids. The recombinant bacmid was transfected into Spodoptera *frugiperda* (Sf9) insect cells according to the manufacturer’s instructions (Life Technologies). The isolated P1 recombinant BV was amplified to generate P2 and P3 virus stocks and then further purified by plaque assay as describe before^16^. To test the expression of Ozz ligase components, Sf9 cells were seeded in 6-well plates (1 × 10^6^ cells/well) in serum-free SFX insect cell medium (Hyclone) and infected with BV. The infected cells were incubated at 27 °C and harvested after 3 days. Aliquots (10 μl) of cell lysates were resolved on SDS–polyacrylamide gels and stained with Coomassie blue Brilliant (BIORAD). No Humans subjects were used in this study.

**Table 1:** Primers used for the generation of CRL5^Ozz^ components.

Gene	Primers	
Ozz	Ozz For	5′ aaggatccgacgctgctgcctccgag 3′
	Ozz Rev	5′ ccgaattctcactcatacttgcagaaatcc 3′
Elongin B	EloB For	5′ cgggatccatggacgtgttcctcatgatccg 3′
	EloB Rev	5′ cggaattctcactgcacggcttgttcattgg 3′
Elongin C	EloC For	5′ gatcggatccatggatggagaggagaaaacctat 3′
	Eloc Rev	5′ gatcggatccaaatttcaactttgattgctatgca 3′
Rbx1	Rbx1 For	5′ taactagtgacgcggcagcgatggatgtgg 3′
	Rbx1 Rev	5′ cctctagactagtgcccatactttt 3′
Cul5	Cul5 For	5′ taggatccatggcgacgtctaatctgttaaa 3′
	Cul5 Rev	5′ ccggatccccatgatattcaaaattatgcc 3′

### Antibodies and reagents

Rabbit anti-Ozz antibody and anti-Alix antibody were prepared as described in^9,10^, respectively. Anti-MyHCemb (2B6) 1:500, was a gift from Dr. N. Rubinstein. Other commercial antibodies included mouse anti-GST 1:500 (UPSTATE), mouse anti-ubiquitin 1:500 (Thermofisher), mouse anti-Elo C 1:300 (BD Biosciences), rabbit anti-Elo B 1:300 (Santa Cruz), rabbit anti-Rbx-1 1:500 (NeoMarkers), rabbit anti-Cul5 1:200 (Santa Cruz).

### Protein expression and purification

*Tni* PRO (*Trichoplusia ni*) (Expression system) cells (1 × 10^6^ cells/ml) were seeded in disposable Erlenmeyer flasks (1000 ml/flask; Corning Life Sciences) and infected with Ozz–EloBC or Cul5–Rbx1 BV. Infected cells were incubated at 27 °C for 72 h in an orbital shaker-incubator (135 rpm). Cells were harvested by centrifugation (1000*g*, 15 min) and resuspended in Tris–HCl lysis buffer (50 mM Tris HCl pH 7.6, 150 mM NaCl, 30 mM Imidazole) and sonicated with 6 pulses for 10 s with a Branson sonicator at setting 3. The cell lysates were centrifuged 2 times at 15,000 rpm for 30 min at 4 °C. Ni-NTA agarose beads (QIAGEN) were spun at 2000 rpm for 5 min and washed with 1 ml of water, spun at 2000 rpm for 5 min, washed once with 2 ml lysis buffer, spun at 2000 rpm for 5 min, then resuspended in 1 ml of lysis buffer (50% slurry), added to the cell lysate supernatant and incubated for 2 h at 4 °C. The lysate was spun at 1000 rpm for 5 min at 4 °C, the resultant pellet was resuspended in 1 ml of washing buffer: (50 mM Tris HCl pH 7.6, 150 mM NaCl and 30 mM Imidazole) and loaded onto an Econo-Pac Chromatography column (BIORAD). The beads were wash twice with 10 ml of washing buffer. The bound proteins were eluted in 0.5 ml Elution buffer (50 mM Tris HCl pH 7.6, 50 mM NaCl, 200 mM Imidazole and 10% Glycerol) and subjected to SDS–polyacrylamide gel analysis stained with Coomassie brilliant blue (BIORAD).

### Reconstitution of CRL5^Ozz^ in vitro by gel filtration

Ozz–EloBC and Cul5–Rbx1 complexes were mixed in a 1:1 ratio and run through a Superose 6 10/300GL gel filtration column (GE Healthcare). The column was equilibrated with 50 mM Tris pH 7.6, 150 mM NaCl. Sample was applied to the preequilibrated column at a flow rate of 0.3 ml/min. The gel filtration fractions (250 μl) were pooled and concentrated in an Amicon Ultra column (Millipore). 14 μl of the concentrated fractions was heat denatured and run on SDS–polyacrylamide gels to determine their constituents.

For calculation of the molecular weight, the column was calibrated with the following protein markers: thyroglobulin, 669 kDa; apoferritin, 443 kDa; β-amylase, 200 kDa; carbonic anhydrase, 29 kDa (BIO-RAD).

### Immunoprecipitation of CRL5^Ozz^

Purified Ozz–EloBC and Cul5–Rbx1 were mixed at a 1:1 ratio and incubated on ice for 1 h. The mixture of the two subcomplexes was resuspended in IP buffer (50 mM Tris HCl pH 7.6, 150 mM NaCl, 500 mM EDTA and 01% NP-40). Gammabind Plus Sepharose beads (GE Healthcare) were washed three times with IP buffer and added to the Ozz ligase and incubated for 1 h. Preclear Ozz ligase was incubated with 2.5 μg of anti Elongin C (BD Bioscience) and 5 μg of Rbx1 (Neomarkers) antibodies for 2 h at room temperature (RT). Samples were immunoprecipitated with Gammabind Plus Sepharose (GE Healthcare) for 1 h a RT. The beads were washed three times with IP buffer and once with IP buffer without detergents. Bound proteins were released by boiling the beads with sample buffer and separated on SDS–polyacrylamide gels under denaturing conditions, followed by SYPRO Ruby Protein Gel Staining (ThermoFisher Scientific).

### Analytical ultracentrifugation

Purified insect cells expressed Ozz E3 sub complexes and reconstituted Ozz-E3 ubiquitin ligase were subjected to sedimentation velocity in a ProteomeLab XL-I analytical ultracentrifuge with a four-hole rotor (Beckman An-60Ti) following standard protocols^17^. Samples in buffer containing 10 mM sodium phosphate, 1.8 mM potassium phosphate pH 7.2, 137 mM NaCl and 0.27 mM KCl were loaded into cell assemblies comprised of double sector charcoal-filled centerpieces with a 12 mm path length and sapphire windows. The buffer density and viscosity were calculated from its composition using the software SEDNTERP (http://www.jphilo.mailway.com/download.htm)^18^.

The partial specific volumes and the molar masses of the proteins were calculated based on their amino acid compositions in SEDFIT (https://sedfitsedphat.nibib.nih.gov/software/default.aspx). The cell assemblies, containing identical sample and reference buffer volumes of 390 µl, were placed in the rotor and temperature equilibrated at rest at 20 °C for 2 h before it was accelerated from 0 to 50,000 rpm. Rayleigh interference optical data were collected at 1-min intervals for 12 h. The velocity data were modeled with diffusion-deconvoluted sedimentation coefficient distributions *c*(*s*) in SEDFIT (https://sedfitsedphat.nibib.nih.gov/software/default.aspx), using algebraic noise decomposition and with signal-average frictional ratio and meniscus position refined with non-linear regression^19^. The s-values were corrected for time and finite acceleration of the rotor and was accounted for in the evaluation of Lamm equation solutions^20^. Maximum entropy regularization was applied at a confidence level of P-0.68.

Two-dimensional size-shape distribution, *c*(*s,f/f*_*0*_) (with the one dimension the *s-*distribution and the other the *f/f*_*0*_-distribution) was calculated with an equidistant *f/f*_*0*_-grid of 0.2 steps that varies from 0.5 to 2.5, a linear *s*-grid from 1 to 20 S with 100 s-values. Tikhonov–Phillips regularization at one standard deviation. The velocity data were transformed to *c *(*s,f/f*_*0*_),* c*(*s*,*M*) and *c*(*s,R*) and distributions with M the molar mass, R the Stokes radius, *f/f*_*0*_ the frictional ratio and *s* the sedimentation coefficient and plotted as contour plots. The color temperature of the contour lines indicates the population of species^21^.

The signal-weighted-average sedimentation coefficient s_w_ provides a measure of species populations in a system. Therefore, signal-weighted-average sedimentation coefficient values, *s*_*w*_, were derived from the integration of all the species from 3 to 7 S of the *c*(*s*) distributions of subcomplex Ozz–EloBC at concentrations 1.8 to 13.3 μM. This measured isotherm of s_w_ as a function of solution composition were then modeled as a reversible monomer–dimer self-association system using SEDPHAT (https://sedfitsedphat.nibib.nih.gov/software/default.aspx). The association scheme was A + A ↔ (A)_2_ with K_D12_ the dimer dissociation constant, A the monomer and (A)_2_ the dimer. Nonlinear least square analysis was performed where the equilibrium association constant, *K*_12_, was optimized in the fit, (*K*_12_ = 1/*K*_D12_)^22^. The errors for this fit represented the 68% confidence interval (CI) using an automated surface projection method^23^. All plots were created in GUSSI (http://www.utsouthwestern.edu/labs/mbr/software/)^24^.

### Analytical glycerol gradient ultracentrifugation—micro-fractionation

A 15–45% glycerol gradient containing 20 mM Tris–HCl pH 7.6, 150 mM NaCl buffer was constructed by layering solutions at decreasing percentages from 45 to 15% glycerol (13 layers, 98 µl each; total volume: 1.30 ml; height: 3.0 cm) in an 11 × 34-mm centrifuge tube. Protein solution (27.5 µl) was layered on top of the gradient followed by 50 µl cold silicon oil to prevent evaporation. The tube was then placed in a pre-cooled bucket and centrifuged for 8 or 12 h at a rotor speed of 55,000 r.p.m. at 4 °C in an Optima TLX preparative ultracentrifuge using a swinging bucket TLS-55 rotor (Beckman Coulter, Fullerton, CA, USA). Deceleration was performed without braking, and the tube was immediately placed on ice. Micro-fractionation of the tube contents was carried out using a BRANDEL automated micro-fractionator equipped with the FR-HA 1.0 block assembly (Brandel, Gaithersburg, MD, USA). The tube was placed in the receptacle, and fractions were removed from the upper surface of the solution by stepwise elevation of the receptacle by a precise increment of height. A total of 27 fractions were collected in a 96-well plate; each fraction was approximately 45 µl in volume, and the bottom fraction was 125 µl^25^. To calculate the molecular weight a mix of the following proteins: apoferritin, 443 kDa (15 μg); β-amylase, 200 kDa (15 μg); Albumin, 66 kDa (15 μg); (SIGMA) were mixed and loaded on top of the glycerol gradient. The markers were centrifuged at the same experimental conditions as the CRL5^Ozz^ and substrates.

### Protein analysis

The protein fractions obtained from the gel filtration column or analytical glycerol gradient ultracentrifugation were analyzed on 4–20% gradient SDS–polyacrylamide gels (BIORAD), the SDS–polyacrylamide gels were stained with SYPRO Ruby protein gel stain (ThermoFisher Scientific). The gels stained were photographed in a Chemidoc MP Image System (BIORAD), and, where appropriate, band densities measured with ImageJ software. Montages were assembled using Adobe Illustrator, and then converted to JPEG files.

### In vitro ubiquitination

The ubiquitination assay was performed by incubating 3.0 µg of a bacterially expressed GST-β-catenin, GST-MyHC_emb_ fragment (1041–1941 a.a.) and GST-Alix with 150 ng (1.2 μM for each 10 μl reaction) of purified recombinant E1 (UBE1-BostonBiochem), 200 ng UbcH5b (11.7 μM for each 10 μl reaction), 1.0 µg (~ 6.4 μM for each 10 μl reaction) CRL5^Ozz^ ubiquitin ligase and 7.5 µg (781.2 μM for each 10 μl reaction) of ubiquitin or ubiquitin K48 mutant (BostonBiochem) in a final volume of 30 µl of ubiquitination buffer (0.05 M Tris–HCl, pH 7.6; 0.01 M MgCl_2_, 0.004 M ATP) for 60 min at 30 °C.

To analyze the ubiquitinated products, the ubiquitination reactions were diluted in 500 μl RIPA buffer (50 mM Tris HCl (pH 7.5), 150 mM NaCl, 1% NP-40, 0.1% deoxycholate, 0.1% SDS, 1 mM EDTA, protease inhibitors and phosphatase inhibitors), immunoprecipitated with 5 µl anti-β-catenin (BD-Bioscience), 20 µl anti-MyHC_emb_ (2B6) or 20 µl anti-Alix (d’Azzo Lab), resolved on 7.5% SDS–polyacrylamide gel, and immunoblotted with anti-ubiquitin (ThermoFisher Scientific), anti-β-catenin, anti-MyHC_emb_ or anti-Alix antibodies.

### Western blotting

Protein concentrations were determined as OD 595, using BSA as standard. 10 µg of soluble protein (100 V, 60 min) was electrophoresed on 12% SDS–polyacrylamide gels or gradient gels (4–20%, BIORAD), and wet-blotted for 3 h at 50 mA. Membranes were probed with specific antibodies at the dilutions listed above, followed by HRP conjugated goat anti-rabbit or anti-mouse IgG (Jackson ImmunoResearch Laboratories). Signals were detected with a West Femto maximum sensitivity substrate kit (Thermo Scientific) on blue films (Midsci).

### Preparation of figures

In the Figures and Supplementary Figures, some images of SDS polyacrylamide gels stained with CBB or SYPRO Ruby, as well as of Western Blots were cropped to display the relevant data (see Supplementary Information file for the uncropped gels or blots). Montages of panels in each Figure were assembled using Adobe Illustrator and converted to TIFF files.

## Results

### Expression and purification of CRL5^Ozz^ components

**Figure 1:**
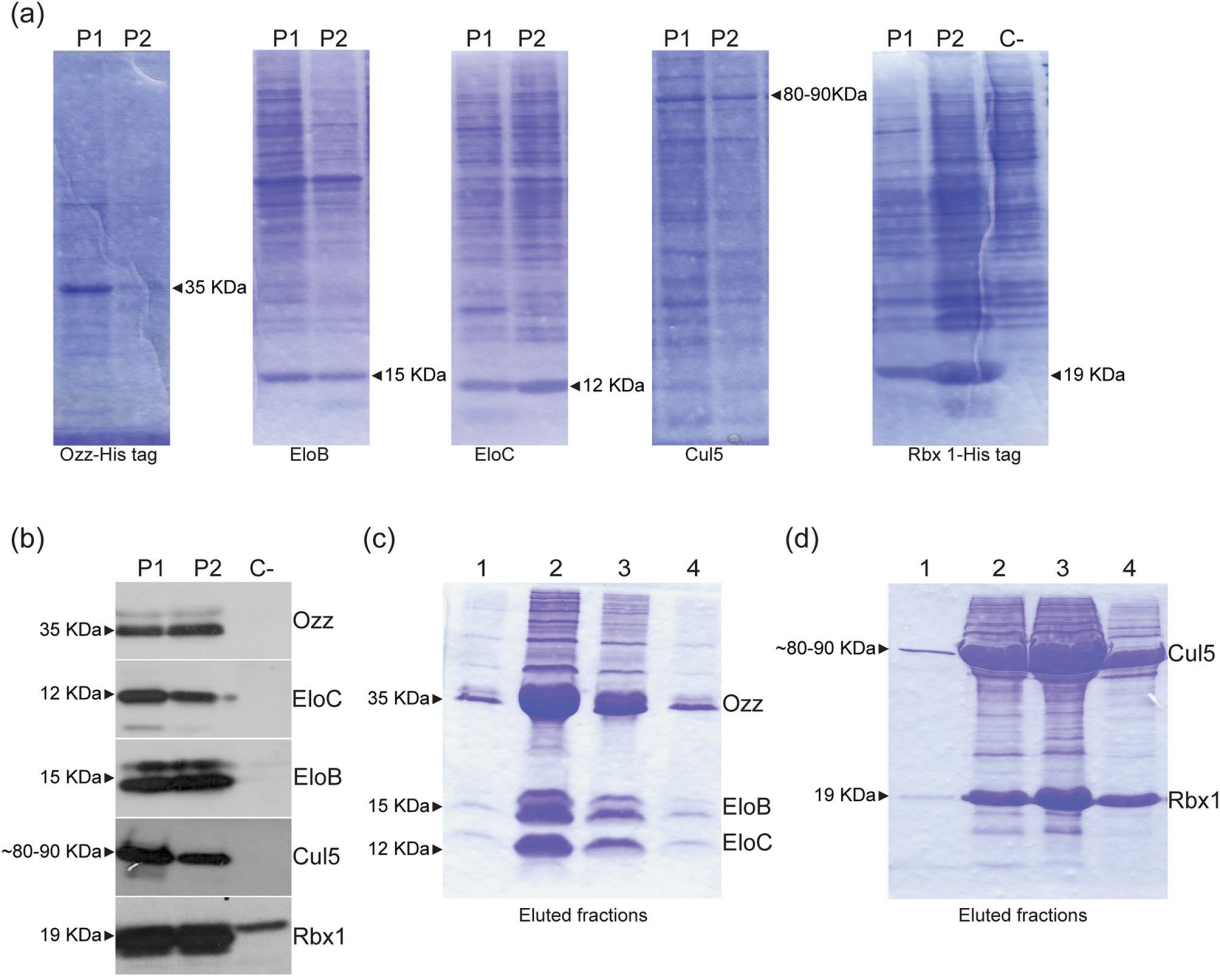
Expression and purification of CRL5^Ozz^ components. (**a**) Coomassie brilliant blue (CBB)–stained gels of baculovirus-produced Ozz (Plaque 1, Plaque 2), Elo B (Plaque 1, Plaque 2), Elo C (Plaque 1, Plaque 2), Cul 5 (Plaque 1, Plaque 2), and Rbx1 (Plaque 1, Plaque 2) from total lysates of individual plaques, compared to mock control (Control). (**b**) Western blot analysis of total lysates overexpressing the individual CRL5^Ozz^ components probed with of anti-Ozz, anti-Elo B, anti-Elo C, anti-Cul5 and anti-Rbx1 specific antibodies. (**c**,**d**) CBB-stained SDS‐polyacrylamide gels of Ni-NTA agarose affinity purified Ozz–EloBC and Cul5–Rbx1 subcomplexes.

To study the assembly of CRL5^Ozz^ in vitro we chose a BV-based system in insect cells to co-express the 5 components of the ligase complex. For this purpose, we first ascertained that all 5 proteins could be expressed at comparable levels. We therefore performed plaque assays of Sf9 insect cells infected separately with the individual BV-constructs, encoding human Ozz, EloB and EloC, Cul5 and Rbx1, and selected for the best expressing clones. All 5 CRL5^Ozz^ components appeared to express at similar levels when tested on Coomassie-stained SDS–polyacrylamide gels (Fig. 1a). Their expression was also validated by Western blot analysis using the corresponding monospecific antibodies (Fig. 1b). To obtain an assembled CRL5^Ozz^ complex, we opted to co-expressed two sets of proteins separately, Ozz–EloBC, and Cul5–Rbx1, rather than co-expressing all 5 components simultaneously that resulted in low yield purification of CRL5^Ozz^ and unequal rate of expression of the individual proteins. For this reason and for further purification of the subcomplexes, two of the overexpressed proteins, Ozz and Rbx1, carried a histidine (His) tag.

To obtain high yield of the overexpressed proteins, we assessed the rate of infection and protein production in two additional insect cell strains, *Tni*PRO and *expres*SF+, and compared them with those obtained with the routinely used Sf9 cells. Co-expression of Ozz–EloBC and Cul5–Rbx1 in *Tni*PRO cells, followed by His tag-purification of the two subcomplexes, showed a twofold higher expression of the purified protein complexes than in *expres*SF+ cells and about 3–4 fold higher expression than in the original Sf9 cells (Fig. 1c,d) These results established *Tni* PRO as the most suitable and reliable insect cell strain to obtain high quantities of the overexpressed subcomplexes. We also tested the optimal buffer composition and pH that afforded the best purification profile and gave a consistent quality and yield of the purified complexes (Supplementary Fig. S1). No differences among the three buffer conditions were observed (Supplementary Fig. S1).

Overall, these results underscore the importance of testing multiple insect cell strains, different buffer compositions and pH to optimize the yield of the co-expressed proteins and to ensure the quality of the final products.

### Ozz–EloBC and Cul5–Rbx1 assemble in vitro into CRL5^Ozz^

**Figure 2:**
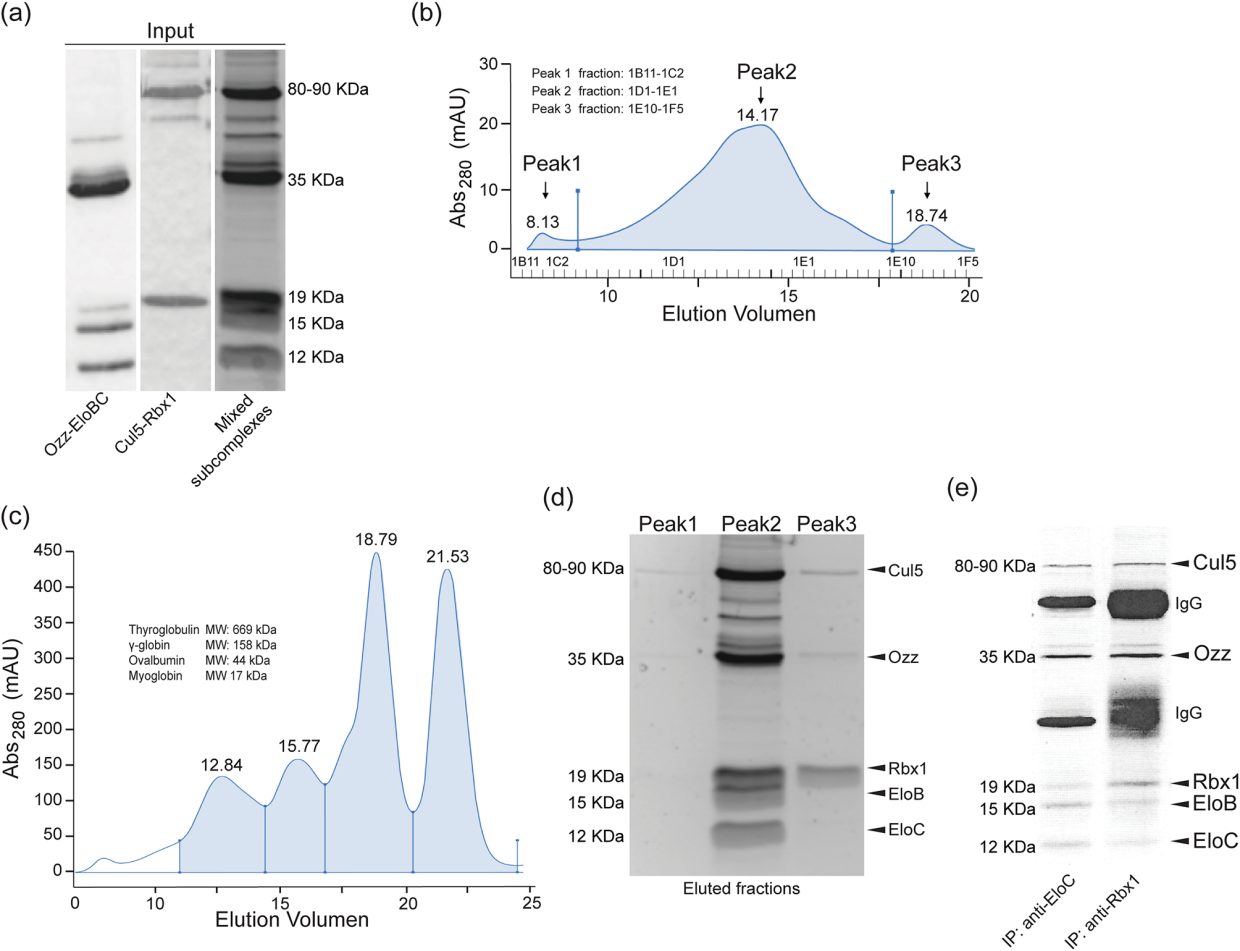
Analysis of CRL5^Ozz^ assembly by gel filtration chromatography and coimmunoprecipitation. (**a**) Affinity purified Ozz–EloBC (~ 60 kDa), Cul5–Rbx1 (~ 100 kDa) and the mixture of the two subcomplexes were visualized on SDS‐polyacrylamide gels stained with SYPRO Ruby. (**b**) CRL5^Ozz^ chromatography profile showing the elution volume and the fractions corresponding to the three major peaks (Peak 1, Peak 2 and Peak 3). (**c**) Superose 6 10/300 size exclusion chromatography markers: thyroglobulin, 669 kDa; apoferritin, 443 kDa; β-amylase, 200 kDa; carbonic anhydrase, 29 kDa. (**d**) The 1:1 mixture was loaded onto a gel filtration chromatography column. Fractions from Peak 1, Peak 2 and Peak 3 were pooled, concentrated and their protein content visualized on SDS‐polyacrylamide gels stained with SYPRO Ruby. All 5 components of CRL5^Ozz^ eluted together mainly in Peak 2 (RV 14.17 = ~ 340 kDa), corresponding to 1D1–1E1 fractions. (**e**) Purified Ozz–EloBC and Cul5–Rbx1 subcomplexes were mixed in a 1:1 ratio and subjected to immunoprecipitation using either anti-EloC or anti-Rbx1 antibodies. Immunoprecipitated samples were analyzed on SDS–polyacrylamide gels stained with SYPRO Ruby. Both antibodies co-immunoprecipitated all CRL5^Ozz^ components, confirming the formation of the assembled complex by mixing the two subcomplexes in vitro.

Next, we asked whether the Ozz–EloBC and Cul5–Rbx1 subcomplexes could assemble in vitro into the 5-component CRL5^Ozz^ complex. For this purpose, we carried out hydrodynamic analyses of the separate and combined subcomplexes using two classical techniques: size-exclusion chromatography and analytical glycerol gradient ultracentrifugation. Both these methods separate complexes based on their molecular weight. For size exclusion chromatography, a 1:1 mixture of the two purified subcomplexes (Fig. 2a) was loaded directly onto a gel filtration column. The chromatography profile showed that the mixture of the two subcomplexes eluted from the column mainly in one broad peak (RV = 14.17 ml), corresponding to molecular weight of ~ 340 kDa (Fig. 2b), as calculated from the elution profile of the protein standards (Fig. 2c). SDS–polyacrylamide gel analysis of the eluted fractions showed that the bulk of all 5 proteins of the complex were resolved together in a single fraction (Peak 2, Fig. 2b,d) and only a small proportion eluted in Peak 3. Based on their size distribution the fraction containing all five components of CRL5^Ozz^ had a calculated molecular weight of ~ 340 kDa (R_V_ = 14.17 ml), suggesting that CRL5^Ozz^ eluted from the column as a dimer (Fig. 2a–d). To prove that CRL5^Ozz^ was reconstituted in vitro, the mixture of Ozz–EloBC and Cul5–Rbx1 subcomplexes was subjected to immunoprecipitation using antibodies against Elongin C or Rbx1. Immunoprecipitated proteins were then separated on SDS–polyacrylamide gels and stained with SYPRO Ruby (Fig. 2e). The results demonstrated that Ozz–EloBC and Cul5–Rbx1 subcomplexes (Fig. 2a) indeed interact with each other and assemble into a 5-component CRL5^Ozz^ (Fig. 2e).

**Figure 3:**
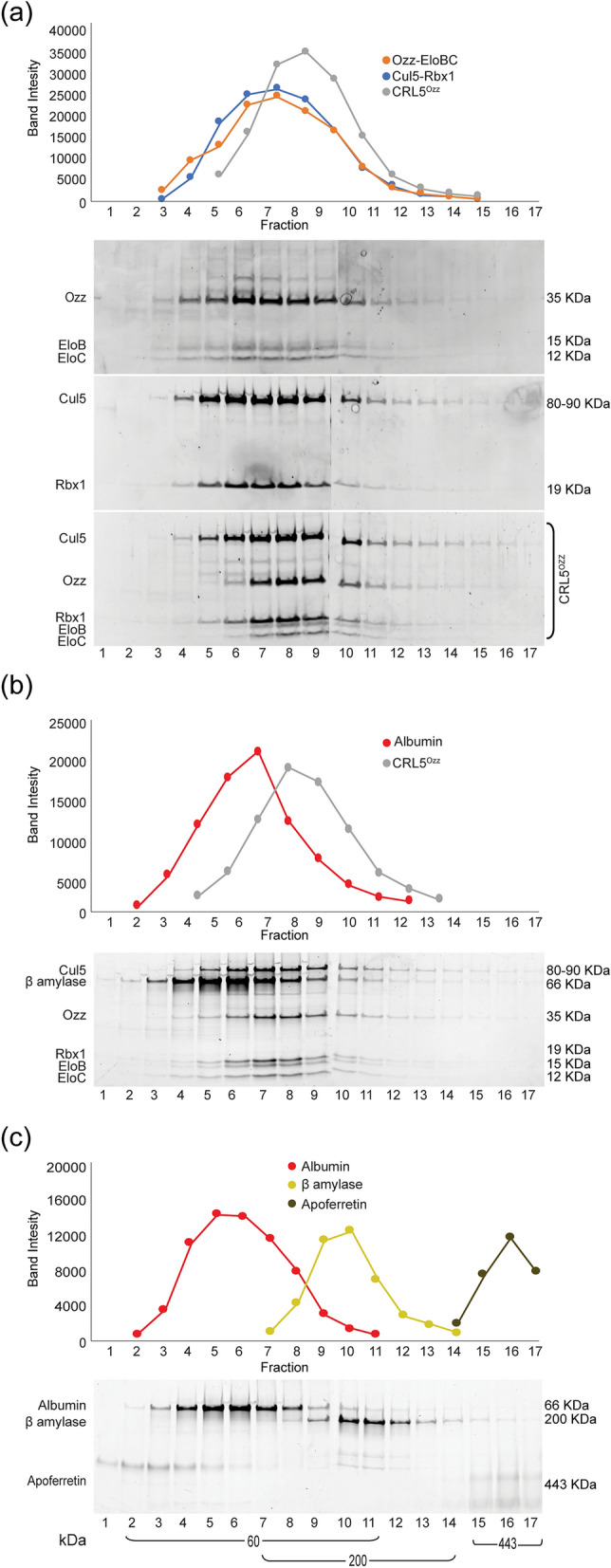
Analysis of CRL5^Ozz^ by glycerol gradient ultracentrifugation microfractionation. (**a**) Sedimentation profiles of the individual Ozz–EloBC and Cul5–Rbx1 subcomplexes as well as the 1:1 mixture of the two (7.5 μg Ozz–EloBC and 7.5 μg Cul5–Rbx1) were obtained by densitometric measurement of band intensity of eluted proteins after glycerol gradient ultracentrifugation. Aliquots of the first 18 fractions of the total 25 fractions collected were analyzed on SDS–polyacrylamide gel stained with SYPRO Ruby. The Ozz–EloBC (~ 60 kDa, upper panel) and Cul5–Rbx1 (~ 100 kDa, middle panel) subcomplexes eluted in fractions corresponding to their calculated molecular weight. The assembled CRL5^Ozz^ elution pattern shifted to fractions corresponding to its monomeric molecular weight (~ 160 kDa, lower panel). (**b**) 15 μg of a CRL5^Ozz^ complex and 15 μg of albumin were mixed and run together on a glycerol gradient. The sedimentation profile of CRL5^Ozz^ was not altered by the presence of albumin. (**c**) Sedimentation profile of Apoferritin (~ 443 kDa), β-amylase (~ 200 kDa) and albumin (~ 60 kDa) protein markers.

We next fractionated the Ozz–EloBC and Cul5–Rbx1 subcomplexes, as well as the assembled CRL5^Ozz^ on glycerol density gradients using ultracentrifugation. All gradients were run simultaneously and under the same conditions. Fractions from each gradient were separated on SDS–polyacrylamide gels and stained with SYPRO Ruby. The fractionation profiles of the complexes and their protein components were generated by densitometric measurements of band intensities (Fig. 3a). Molecular weights were calculated based on the fractionation patterns of several gel filtration markers (Fig. 3c). Using this method, we found that the bulk of Ozz–EloBC is contained in fraction 3 to fraction 10, trailing minorly until fraction 12. The fractionation curve for this subcomplex indicated a size range of ~ 60–443 kDa (Fig. 3a, upper panel). The Cul5–Rbx1 subcomplex was fractionated in a nearly identical pattern and similar molecular weight range (Fig. 3a, middle panel). However, the mixture of Ozz–EloBC and Cul5–Rbx1 gave a different fractionation profile with all 5 components eluting together in fractions 5–11 (Fig. 3a, lower panel). The peak for CRL5^Ozz^ was in fractions 7–9, and trailing until fraction 17, showing a clear shift in molecular weight compared to the individual subcomplexes (Fig. 3a). These results demonstrate that, by mixing the two subcomplexes, the 5-protein components sedimented together in fractions corresponding to sizes of ~ 220–443 kDa (Fig. 3a,c), containing the assembled CRL5^Ozz^ that again appeared in part dimeric (Fig. 3a). We used albumin as internal control to show that this protein did not shift in molecular weight in presence of CRL5^Ozz^, indicating no physical interaction between this protein and the ligase (Fig. 3b).

### Analytical ultracentrifugation analysis of CRL5^Ozz^ and its subcomplexes

**Figure 4:**
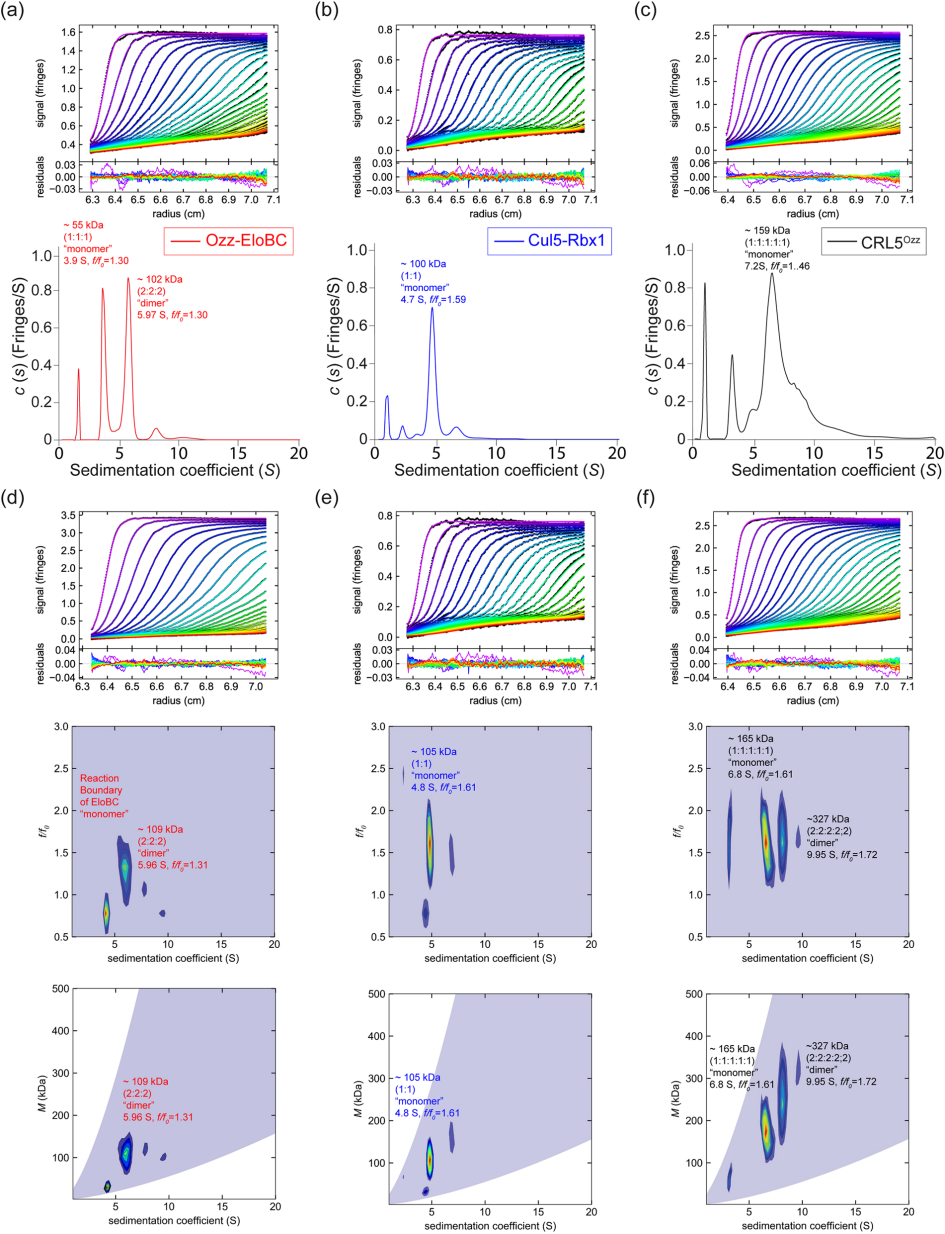
Sedimentation velocity—AUC analysis of Ozz–EloBC, Cul5–Rbx1, and CRL5^Ozz^ complex. Panels (**a**–**c**) Top panel: Fringe displaced sedimentation velocity profiles (fringes vs radius), with superimposed solid lines showing the best fit to the model, and below residuals of the fits. Bottom panel: display of the continuous sedimentation coefficient distribution *c*(*s*) plots of Ozz–EloBC (red line), Cul5–Rbx1 (blue line), and a mixture of the former two complexes (black line); (**d**–**f**) Top panel: fringe displaced sedimentation velocity profiles (fringes vs radius), with superimposed solid lines showing the best fit to the model, and below residuals of the fits. Middle panel: Contour plots (heat maps) of the two-dimensional size-and-shape distributions, *c*(*s,f/f*_*0*_) and Bottom panel: Molar mass distributions *c*(*s,M*), of Ozz–EloBC, Cul5–Rbx1 and a mixture of Ozz–EloBC and Cul5–Rbx1, respectively. The experiments were conducted in 137 mM NaCl, 0.27 mM KCl, 10 mM Na2HPO4 and 1.8 mM KH2PO4 pH 7.2 buffer at 20 °C and at a rotor speed of 50,000 rpm. The s-, *f/f*_*0*_ and *M*-values of the protein complexes are listed in Tables 2 and 3.

We next sought to compare additional hydrodynamic properties of the two separate Ozz–EloBC (Table 2, Fig. 4a) and Cul5–Rbx1 (Table 2, Fig. 4b) subcomplexes, and the reconstituted CRL5^Ozz^ (Table 2, Fig. 4c).

**Table 2:** Summary of results of the velocity *c*(*s*) analysis of Ozz–EloBC, Cul5–Rbx1 and CRL5^Ozz^ (Ozz–EloBC + Cul5–Rbx1) complex in 10 mM sodium phosphate, 1.8 mM potassium phosphate pH 7.2, 137 mM NaCl and 0.27 mM KCl buffer at 20 °C.

Sample	mg/ml^a^	*s*_*20*_ (Svedberg)^b^	*s*_*20,w*_ (Svedberg)^c^	*M* (Da)^d^	*f*/*f*_*0*_^e^
Ozz–EloBC	0.35	3.83 (35%)	3.98	55,325 (57,285)	1.30
		5.75 (53%)	5.97	101,738 (114,571)	1.30
Cul5–Rbx1	0.21	4.70 (69%)	4.88	104,744 (103,212)	1.63
CRL5^Ozz^	0.75	3.52 (8%)	3.66	57,791	1.46
		5.09 (6%)	5.28	100,260	1.46
		6.93 (55%)	7.20	159,245 (160,497)	1.46
		9.02 (19%)	9.38	235,243	1.46
		11.96 (5%)	12.42	358,422 (320,992)	1.46

^a^Total concentration of sample in mg/ml.

^b^Sedimentation coefficient taken from the ordinate maximum of each peak in the best-fit *c*(*s*) distribution at 20 °C with percentage protein amount in parenthesis. Sedimentation coefficient (*s*-value) is a measure of the size and shape of a protein in a solution with a specific density and viscosity at a specific temperature.

^c^Standard sedimentation coefficient (*s*_*20,w*_-value) in water at 20 °C.

^d^Molar mass values (M) taken from the *c*(*s*) distribution that was transformed to the *c*(*M*) distribution with theoretical values in parenthesis. The Ozz–EloB-C preparation shows monomer and dimer.

^e^Best-fit weight-average frictional ratio values (*f/f*_*0*_)_*w*_ taken from the *c*(*s*) distribution.

We first evaluated the sedimentation coefficient distribution profiles, *c*(*s*), of the individual Ozz–EloBC and Cul5–Rbx1 subcomplexes and then that of the reconstituted CRL5^Ozz^ (Table 2, Fig. 4). At 0.35 mg/ml the *c*(*s*) distribution profile of Ozz–EloBC consisted of two major separate peaks, each representing dissimilar sized species, indicating that Ozz–EloBC assembled into oligomers of different masses (Table 2, Fig. 4a). One of the two major peaks had a sedimentation value, *s*_*20,w*_, of 5.70 S, corresponding to molar mass of 101,738 Da, close to the theoretical molecular weight of the 2:2:2 dimer complex (Table 2, Fig. 4a); the other major peak had an *s*_*20,w*_-value of 3.98 S, corresponding to a molar mass of 55,325 Da, close to the theoretical molecular weight of the 1:1:1 monomer complex (57,591 Da) (Table 2, Fig. 4a). The best-fit weight-average frictional value of 1.30 obtained from the analysis is indicative of a slightly extended globular shape of the protein complex. In contrast, the sedimentation analysis of Cul5–Rbx1 (Table 2, Fig. 4b) of similar concentration, 0.21 mg/ml, showed only one major peak with an *s*_*20,w*_-value of 4.88 S, that corresponds to a molar mass of 104,744 Da, close to the theoretical molecular weight of a 1:1 protein complex (103,212 Da) (Table 2, Fig. 4b). The best-fit weight-average frictional ratio of 1.63 suggests that the molecular shape of this subcomplex is extensively elongated.

Next, we tested the sedimentation distribution profile of CRL5^Ozz^ (Table 2, Fig. 4c) in a 1:1 mixture of Ozz–EloBC and Cul5–Rbx1. Remarkably, the *c*(*s*) profile of the reconstituted CRL5^Ozz^ showed a distribution of new peaks with higher *s*-values that corresponded to complexes with higher molar masses than those of the individual subcomplexes (Table 2, Fig. 4c). This result indicated that the Ozz–EloBC and Cul5–Rbx1 mixture has a different sedimentation profile than the separate Ozz–EloBC or Cul5–Rbx1 subcomplexes (Fig. 4a,b). The different peaks obtained with the individual Ozz–EloBC or Cul5–Rbx1 subcomplexes were present, but at a much reduced amount in the mixture, indicating that the two subcomplexes assembled at least in part into a five-protein CRL5^Ozz^ multimeric complex. From this *c*(*s*) distribution one major peak (55% of total protein) was distinguishable from the rest with an *s*_*20,w*_-value of 7.20 S, that corresponded to molar mass of 159,245 Da, close to the theoretical molecular weight of an 1:1:1:1:1 five-protein CRL5^Ozz^ (160,497 Da) (Table 2, Fig. 4c). The best-fit weight-average frictional ratio of 1.46 indicates a molecular shape that is elongated.

Analysis of the same sedimentation velocity data with the two-dimensional size-shape distribution model, *c*(*s,f/f*_*0*_), yielded similar results (Table 3, Fig. 4d–f). The Ozz–EloBC subcomplex showed an *s*_*20,w*_-value of 6.19 S, a molar mass of 108,998 Da, close to the 106,424 Da dimer mass, and a frictional ratio of 1.31, indicating a folded, slightly extended, globular molecular shape (Table 3, Fig. 4d). The Cul5–Rbx1 subcomplex yielded an *s*_*20,w*_-value of 5.00 S, a molar mass of 105,821 Da, close to the theoretical molecular weight of 103,212 Da, and a frictional ratio of 1.61, indicating an elongated molecular shape (Table 3, Fig. 4e). The Ozz–EloBC and Cul5–Rbx1 mixture showed the presence of a peak (48% of total proteins) with an *s*_*20,w*_-value of 6.85 S and a frictional ratio value of 1.61 that corresponded to a molar mass of 165,688 Da, close to the five-protein 1:1:1:1:1 CRL5^Ozz^ molecular weight (160,497 Da) (Table 3, Fig. 4f). Again, the large frictional ratio suggests an elongated molecular shape. However, CRL5^Ozz^ also formed complexes with higher stoichiometry; a peak (8% of total proteins) with an *s*_*20,w*_-value of 9.95 S and a frictional ratio value of 1.72 that corresponded to a molar mass of 327,193 Da, close to dimeric CRL5^Ozz^ molecular weight of 320,992 Da. The frictional ratio of this putative dimeric complex of 1.72 indicates a possible partial long-end-to-long-end association of the monomers into the dimer (Table 3, Fig. 4f). By contrast, a similar analysis of the simultaneously co-expressed and purified Ozz + EloB + EloC + Cul5 + Rbx1 complex, displayed several peaks with a *c*(*s*) profile, which ranged from 6 to 15 S, indicating the presence of high molar mass species that were most probably complexes with different stoichiometries than those of the “monomer” or “dimer” reconstituted CRL5^Ozz^ (Supplementary Fig. S2a,b and Supplementary Tables S1, S2).

**Table 3:** Best-fit values and estimates of the 2D *c*(*s,f/f*_*0*_) analyses of Ozz–EloBC and Cul5–Rbx1, and CRL5^Ozz^ (Ozz–EloBC + Cul5–Rbx1) in 10 mM sodium phosphate, 1.8 mM potassium phosphate pH 7.2, 137 mM NaCl and 0.27 mM KCl buffer at 20 °C.

Sample	mg/ml^a^	*s*_*w*_ (Svedberg)^b^	*s*_*20,w*_ (Svedberg)^c^	*M* (Da)^d^	(*f/f*_*0*_)_*w*_^e^	*Rs*^f^
Ozz–EloBC	1.00	5.96 (52%)	6.19	108,998	1.31	4.25
Cul5–Rbx1	0.21	4.82 (59%)	5.00	105,821	1.61	5.10
CRL5^Ozz^	0.75	6.66 (48%)	6.85	165,688	1.61	4.45
		8.12 (18%)	8.43	245,076	1.67	
		9.58 (8%)	9.95	327,193	1.72	7.85
		3.16 (7%)	3.28	60,144	1.68	

^a^Total concentration of sample in milligram per milli liter.

^b^Weight-average sedimentation coefficient *s*_*w*_ calculated from the 2D *c*(*s,f/f*_*0*_) model with percentage protein amount of total protein in parenthesis.

^c^Standard sedimentation coefficient (*s*_*20,w*_-value) in water at 20 °C.

^d^Estimated molar mass calculated from (*s*_*w*_*,f/f*_*0*_) from the 2D *c*(*s,f/f*_*0*_) model.

^e^Weight-average frictional ratio (*f/f*_*0*_)_*w*_ calculated from the 2D *c*(*s,f/f*_*0*_) model.

^f^Stokes radius (nm) obtained from contour plot of the *c*(*s,M*) distribution.

**Figure 5:**
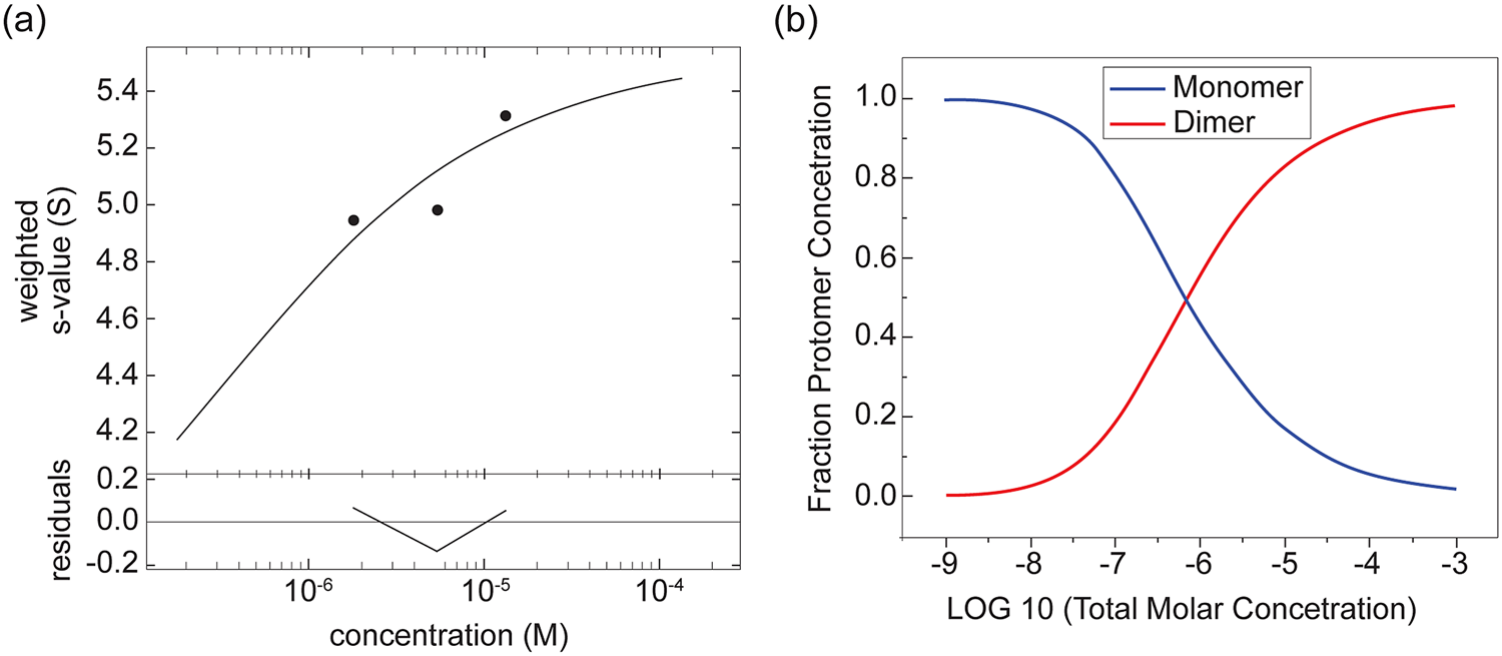
Sedimentation velocity—analytical ultracentrifugation analysis, s_w_ isotherm of Ozz–EloBC. (**a**) The best-fit isotherm of the signal-weight-average s-values, s_w_, obtained by integration of *c*(*s*) distributions of Ozz–EloBC over the s-range of 3 and 7 S for each loading concentration in a dilution series. The solid line is the fitted isotherm to a reversible monomer–dimer self-association model with the best-fit dissociation constants K_D12_ value as well as confidence intervals and the root mean square deviation of the fits at all the concentrations listed (Table 4). (**b**) The species population plots of fraction protomer concentration *vs* log total concentration (Molar) with the amounts of monomer and dimer at specific concentrations determined by the K_D12_ value of the self-association model are also shown.

Because Ozz–EloBC can form a dimer, we also investigated the concentration dependent self-association of this subcomplex (Table 4, Fig. 5a,b). At relatively high concentrations this subcomplex formed a dimer. Therefore, the signal-weighted-average sedimentation coefficient, s_w_, that provides a measure of species populations in a system, was plotted against concentration (Fig. 5a). The dimer dissociation constant value, *K*_*D12*_ obtained from this analysis was 0.70 [0.43, 1.07] µM with the confidence interval, (CI), in parenthesis (Table 4, Fig. 5b). This observation suggests that the Ozz–EloBC subcomplex forms dimers, most probably via an Ozz–Ozz interaction, and strengthens the idea that the Ozz–EloBC and Cul5–Rbx1 mixture can form not only a 1:1:1:1:1 complex but also a dimeric CRL5^Ozz^.

**Table 4:** AUC-SV: summary of the isotherm sedimentation velocity analysis of Ozz–EloBC in 10 mM sodium phosphate, 1.8 mM potassium phosphate pH 7.2, 137 mM NaCl and 0.27 mM KCl buffer at 20 °C. Model: monomer–dimer self-association.

Sample^a^	Conc (µM)^a^	K_D12_ (µM)^b^	RMSD^c^
Ozz–EloBC	13.3, 5.3, 1.8	0.70 [0.43, 1.07]	0.0930

^a^Total concentration of the integrated monomer–dimer *c*(*s*) peaks.

^b^Dissociation constant K_D12_ of the monomer–dimer self-association reaction. For the isotherm signal‐weighted-average sedimentation coefficient values were obtained by integration of the *c*(*s*) distributions between 3 and 7 S at various concentrations^17,22^.

The *s*‐values for monomer and dimer, *s*_1_ and *s*_2_ (3.82 S and 5.75 S) were fixed while the *K*_12_ the equilibrium association constant was optimized in the fit (Theoretical molar mass: 57,285 Da). Errors of the constant represent the 68% confidence interval (CI) using an automated surface projection method^23^.

^c^Root mean square deviation of the fit, units in fringes for the reversible dimer formation.

### CRL5^Ozz^ physically associates with its substrates in vitro

**Figure 6:**
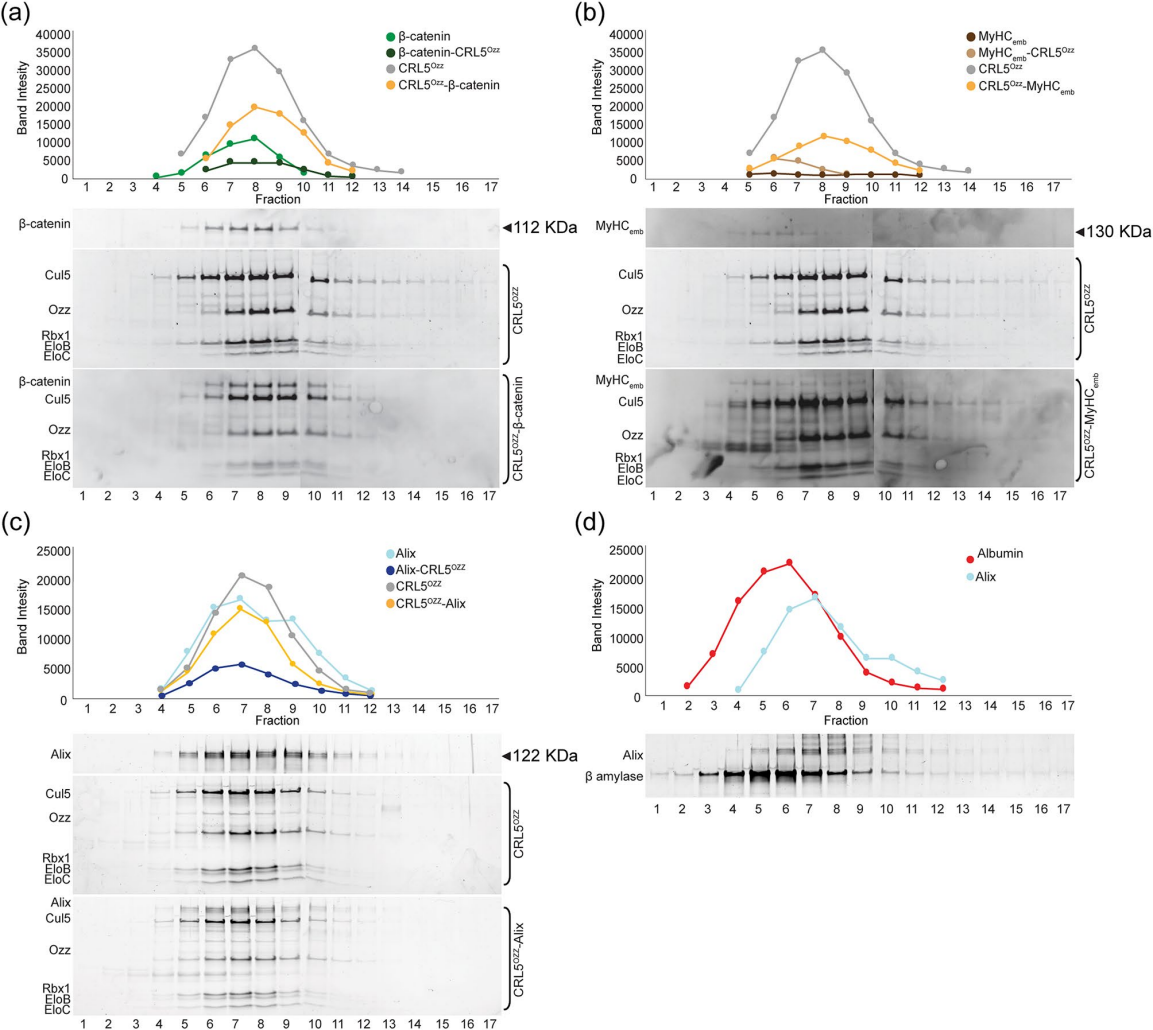
Glycerol gradient ultracentrifugation microfractionation analysis of CRL5^Ozz^ and its substrates. Assembled CRL5^Ozz^ mixed with purified preparations of (**a**) GST- full length β-catenin, (**b**) GST-MyHC_emb_ fragment (1041–1941 a.a.) and (**c**) GST-full length Alix was fractionated from a post-ultracentrifuged glycerol gradient (8 h). Aliquots of each fraction were separated on SDS–polyacrylamide gels and their protein content visualized on gels stained with SYPRO Ruby. The densitometric measurement of band intensity of the proteins in each fraction showed a shift to a higher molecular weight when CRL5^Ozz^ was mixed with either of its substrates, compared to the molecular weights of CRL5^Ozz^ or its individual substrates: CRL5^Ozz^–β-catenin (~ 272 kDa), CRL5^Ozz^–MyHC_emb_ fragment (1041–1941 a.a.) (~ 280 kDa) or CRL5^Ozz^–Alix (~ 282 kDa). (**d**) Sedimentation analysis of Alix mixed with albumin as internal control. The fractions were loaded on an SDS–polyacrylamide gel and stained SYPRO Ruby. Alix and albumin were visible in fractions 2–12 and the profile of either protein was not altered in presence of the other.

To determine whether the reconstituted ligase complex could recognize and bind to its substrates**,** the assembled CRL5^Ozz^ was mixed in vitro with purified preparations of three substrates, glutathione S-transferase (GST)-tagged-β-catenin (~ 112 kDa), GST-MyHC_emb_ fragment (1041–1941 a.a.) (~ 130 kDa) and GST-Alix (~ 122 kDa) (Fig. 6). Assembled CRL5^Ozz^ alone or combined with each of the substrates, as well as the three substrates by themselves were subjected to glycerol gradient ultracentrifugation combined with microfractionation, and proteins were visualized on SDS–polyacrylamide gels stained with SYPRO Ruby (Fig. 6). Also, in this case, all gradients were run simultaneously and under the same conditions. Analyses of individual fractions revealed that GST-β-catenin was detected in fractions 5–10 (Fig. 6a, upper panel), while, combined with CRL5^Ozz^, in fractions 6–12 (Fig. 6a, lower panel), showing a clear shift in its molecular weight. CRL5^Ozz^ was eluted in a nearly identical pattern to GST-β-catenin, an indication that CRL5^Ozz^ co-migrated with GST-β-catenin (Fig. 6a, lower panel). Remarkably, in presence of β-catenin CRL5^Ozz^ sedimented in the same fractions as its substrate without trailing to lower molecular weight fractions as it did without the substrate (Fig. 6a). A similar protein distribution profile was observed when CRL5^Ozz^ was subjected to ultracentrifugation with either GST-MyHC_emb_ (Fig. 6b) or GST-Alix (Fig. 6c). We used albumin as internal control to show that the CRL5^Ozz^ substrate did not change its sedimentation profile in presence of an unrelated protein (Fig. 6d). Similar sedimentation profiles were observed when samples were ultracentrifuged for a longer period (12 h instead of 8 h) (Supplementary Fig. S3a–f).

Based on the molecular profiles in presence or absence of the substrates, we can infer that CRL5^Ozz^ interacts with each of them as a monomer. These results indicate that CRL5^Ozz^ assembles in vitro and retains its ability to recognize and physically interact with each of its substrates (Fig. 6).

### Purified CRL5^Ozz^ interacts and ubiquitinates substrates in-vitro

**Figure 7:**
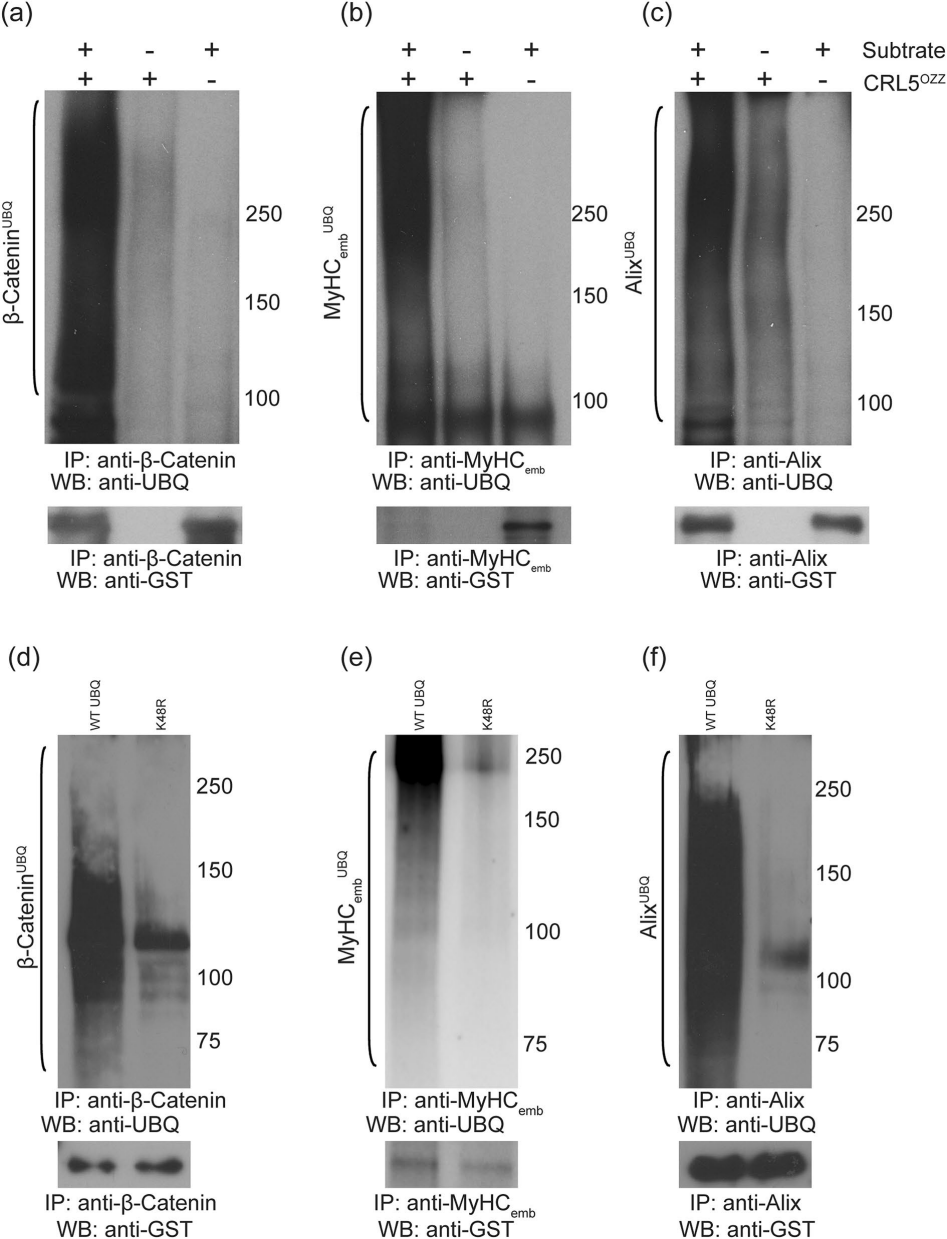
In vitro ubiquitination of recombinant β-catenin, MyHC_emb_ and Alix mediated by CRL5^Ozz^. (**a**,**d**) GST-tagged-β-catenin, (**b**,**e**) MyHC_emb_ fragment (1041–1941 a.a.) and (**c**,**f**) Alix was incubated with CRL5^Ozz^ and either native ubiquitin or mutant Ub (K48R). In the presence of native ubiquitin and CRL5^Ozz^, β-catenin or MyHC_emb_ or Alix were efficiently ubiquitinated. If the assay was performed in the presence of Ub K48R, the ubiquitination was reduced to background levels, demonstrating that CRL5^Ozz^ polyubiquitinated β-catenin, MyHC_emb_ and Alix. (**a**–**c**) CRL5^Ozz^ efficiently ubiquitinated β-catenin, MyHC_emb_ and Alix (lane 1). The specificity of the reaction was confirmed by omitting either the substrate (lane 2) or the CRL5^Ozz^ complex (lane 3) from the ubiquitination assay, which significantly reduced the Ub-substrate products. (**d**–**f**) In the presence of native ubiquitin, CRL5^Ozz^ efficiently ubiquitinated its substrates (lane 1). Instead, by using K48R Ub mutant in the ubiquitination reaction, the ubiquitination of the substrates was significantly reduced (lane 2).

Having purified and reconstituted CRL5^Ozz^, we wanted to ascertain whether it could promote the in vitro ubiquitination of its substrates. Therefore, we incubated purified GST-β-catenin (Fig. 7a), GST-MyHC_emb_ (Fig. 7b) and GST-Alix (Fig. 7c) with the purified CRL5^Ozz^ together with E1, E2 and ubiquitin. Ubiquitinated forms of Ozz substrates were detected only in the presence of CRL5^Ozz^ (Fig. 7a–c). Omission of the ligase or any of the components from the reaction mixtures prevented ubiquitination of the substrates. Lastly, to discern whether CRL5^Ozz^ complex promoted mono, multi or polyubiquitination of its substrates, we performed in vitro ubiquitination assays with either wild type ubiquitin or a ubiquitin mutant carrying K48R amino acid substitution that abrogates the formation of polyubiquitin chains. Polyubiquitination of Ozz substrates occurred only when we used the non-mutated form of ubiquitin in our ubiquitination reaction (Fig. 7d–f). Altogether, these results indicate that Ozz functions in vitro as the substrate-recognition component of CRL5^Ozz^, which recruits and polyubiquitinates β-catenin, MyHC_emb_ and Alix.

## Discussion

CRLs constitute one of the largest family of E3 ligases, which are conserved among species^26,27^. Their pivotal role in cell physiology and homeostasis is evidenced by the pathogenic effects of their impaired or deregulated activity in human diseases, like cancer and neurodegenerative diseases^28,29^. To date, only a hand full of CRL complexes have been described that provide information of their protein components and how they are structurally organized^28,30–33^**.** This is because high expression of their individual full-length proteins, and their reconstitution into active ligase complexes have been difficult to achieve. In addition, despite that over 400 CRL members have been identified, for most of them the natural substrates are still unknown^26,27^.

Here we describe the production and purification of CRL5^Ozz^, a member of the CRL family of ubiquitin ligases that is specific for striated muscle and is involved in myofibrillogenesis and myofiber differentiation^1,9,10^. Within the complex, the scaffold protein Ozz is the substrate-recognition component^1,9,10^. Ozz embeds two adjacent substrate recognition domains, NHR1 and 2, that the protein shares with the drosophila single chain ubiquitin ligase, Neur^15,34^.

Previous biophysical studies on the structural assembly of CRL complexes demonstrated that these multi-subunit Cullin-RING ligases function as monomers or dimers^35,36^. These structural configurations of CRL complexes might be necessary for high avidity binding to the substrates and/or for the acquisition of the optimal stoichiometry for substrate recognition^26^. In two prototypical E3 ubiquitin ligases, CRL2^VHL^ and SCF^FBW7^, the interface that drives dimerization was shown to be mediated by the adaptor proteins VHL and FBW7, respectively^36–38^. Our biophysical analysis of Ozz–EloBC showed that the complex exists both as monomer and dimer, a characteristic that is not shared by the Cul5–Rbx1 complex. The latter observation suggests that the interface for the CRL5^Ozz^ dimer is provided by the Ozz–EloBC complex, and more specifically by Ozz itself. Ozz contains two NHR domains forming the bulk of the protein (amino acids 23–244 out of 285). These domains mediate protein–protein interaction, as demonstrated for the NHR domains of the E3, Neur, and are crucial for the oligomerization of this ligase^15,39^. These authors proposed that the NHR domains of Neur might form an intramolecular structure that regulates its substrate recognition and ubiquitination activity^39^. By analogy with Neur, the NHR domains in Ozz may promote substrate recognition as well as CRL5^Ozz^ dimerization, the latter configuration being abrogated by the presence of the substrate.

Crystal structure studies of the CRL2^VHL^ emphasized the importance of the substrates for the stabilization of the ligase^28^. These authors showed that CRL2^VHL^ cannot form crystals in absence of a 19-mer peptide of its substrate HIF-1α, and reasoned that the substrate maybe required to confer stability to the conformational arrangement of the ligase that facilitates crystallization^28^. This finding suggests that CRL type ligases are highly flexible and acquire more than one structural orientation to accommodate different substrates. As it is the case for other E3’s^40,41^, CRL5^Ozz^ targets multiple substrates located in different cellular compartments. It is, therefore, conceivable that individual Ozz substrates promote conformational rearrangements of CRL5^Ozz^ to stabilize the ligase for optimal delivery of the substrates to the proteasome. However, the exact stoichiometry of CRL5^Ozz^ is still unknown, and further work is needed to define its ultrastructural architecture and the exact mechanism(s) of substrate recognition.

## Supplementary Information

Supplementary Figure S1.

Supplementary Figure S2.

Supplementary Figure S3.

Supplementary Information.

Supplementary Table S1.

Supplementary Table S2.
