# Supplementary figures and images for: Biophysical and functional study of CRL5Ozz, a muscle specific ubiquitin ligase complex
Source: Sci Rep. 2022 May 12;12:7820. doi: 10.1038/s41598-022-10955-w (PMC9098882; doi:10.1038/s41598-022-10955-w)

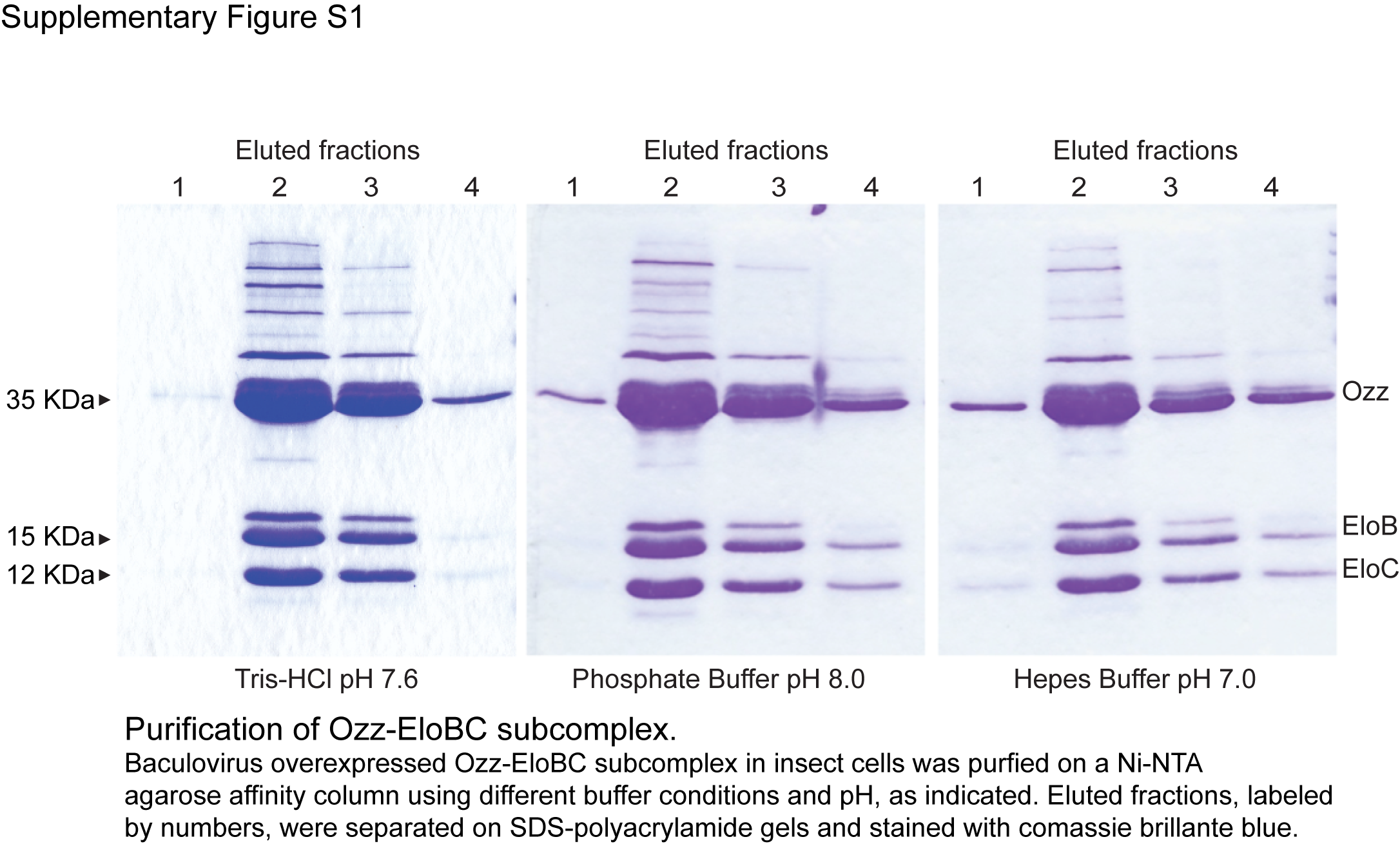

Supplement: Supplementary file 1 — Supplementary Figure S1. [file 41598_2022_10955_MOESM1_ESM.tif]

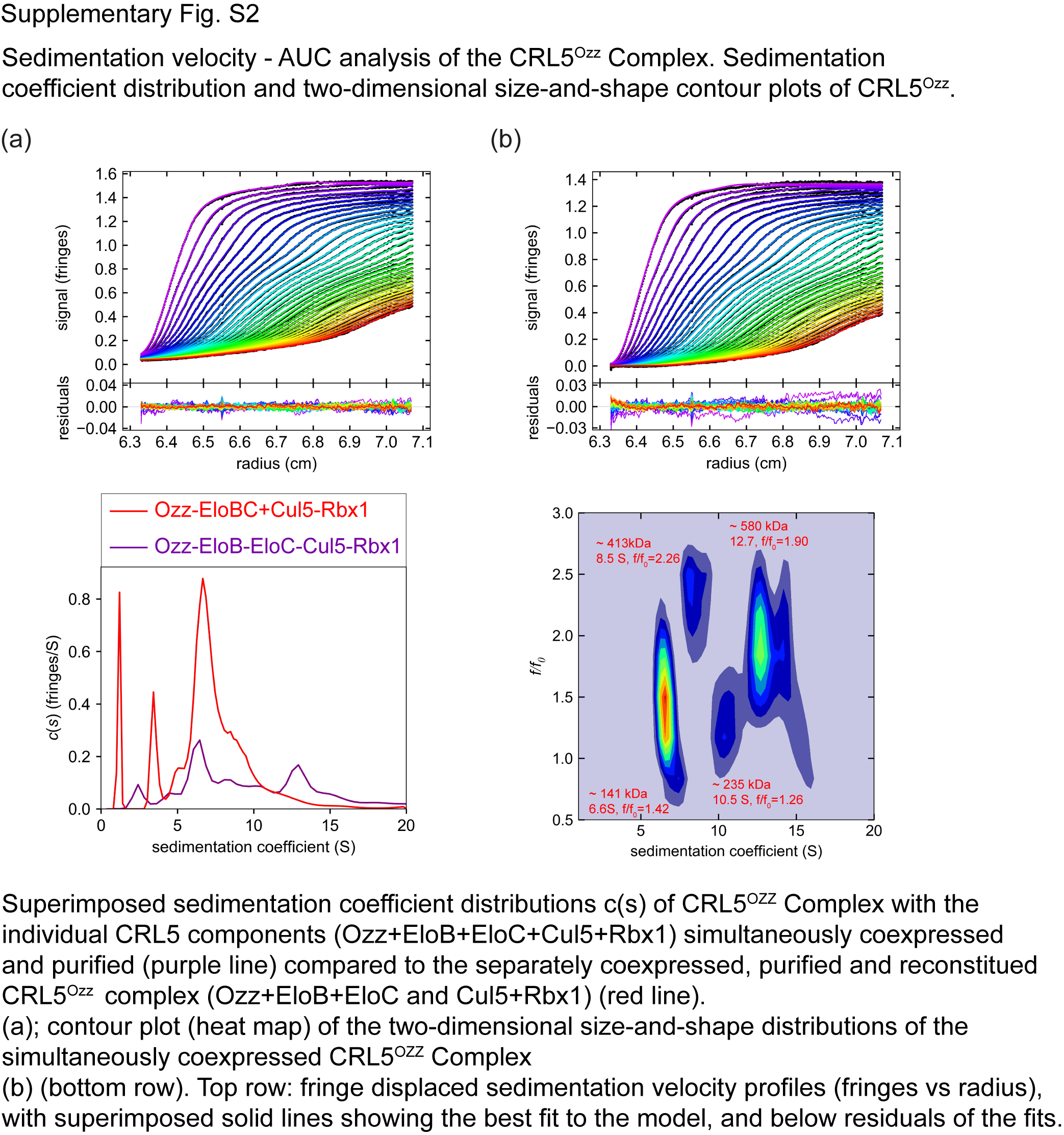

Supplement: Supplementary file 2 — Supplementary Figure S2. [file 41598_2022_10955_MOESM2_ESM.tif]

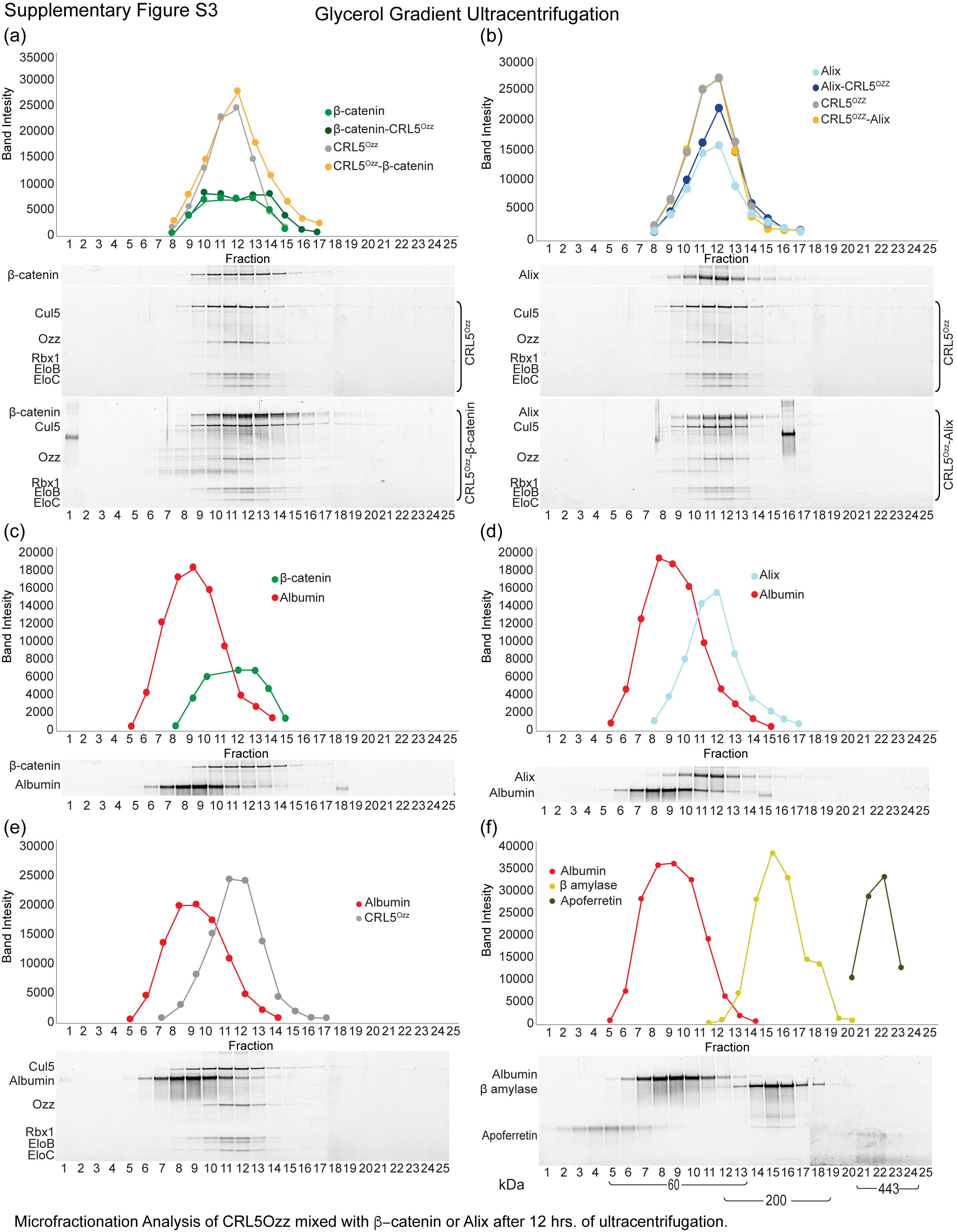

Supplement: Supplementary file 3 — Supplementary Figure S3. [file 41598_2022_10955_MOESM3_ESM.tif]

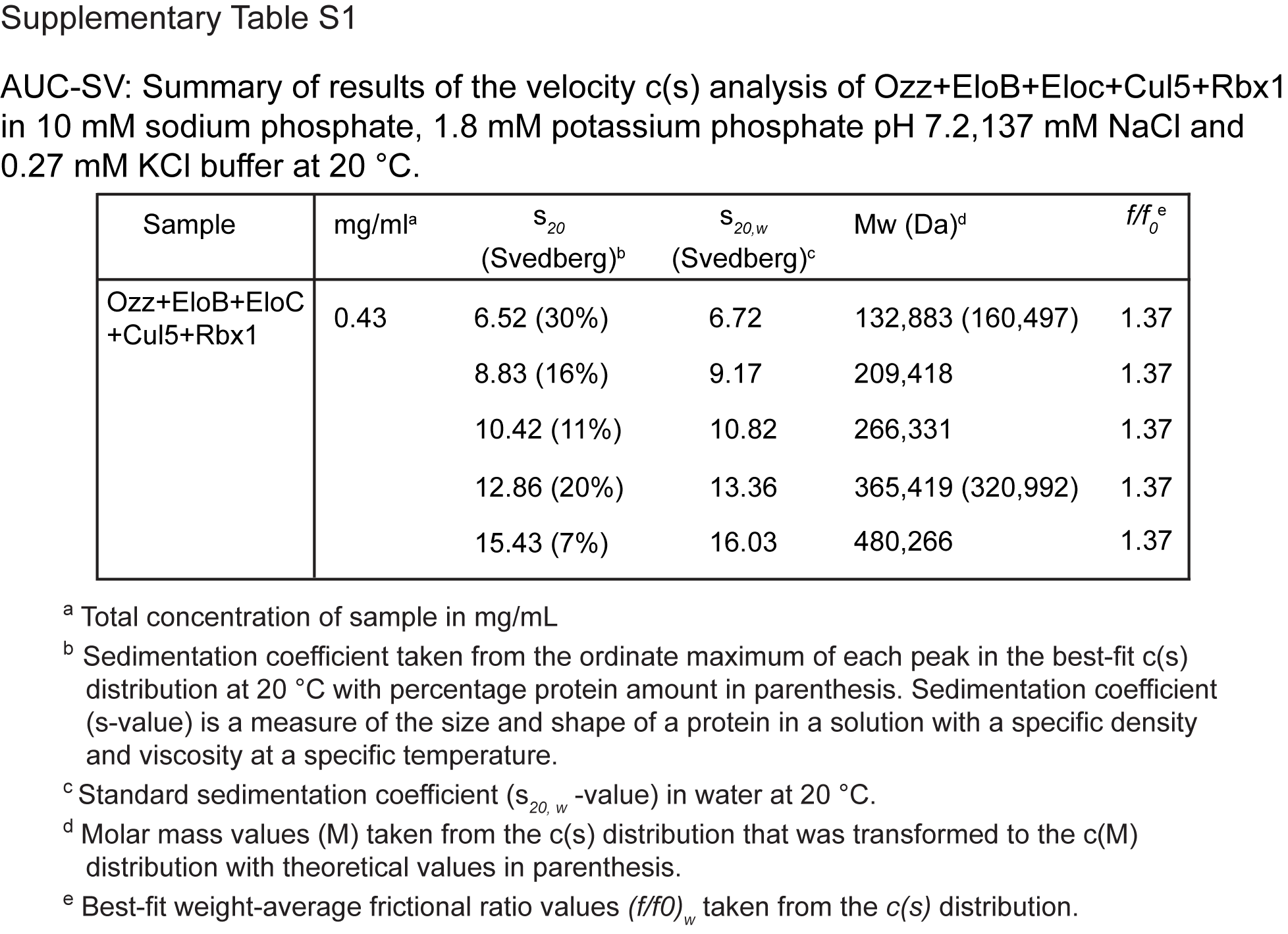

Supplement: Supplementary file 5 — Supplementary Table S1. [file 41598_2022_10955_MOESM5_ESM.tif]

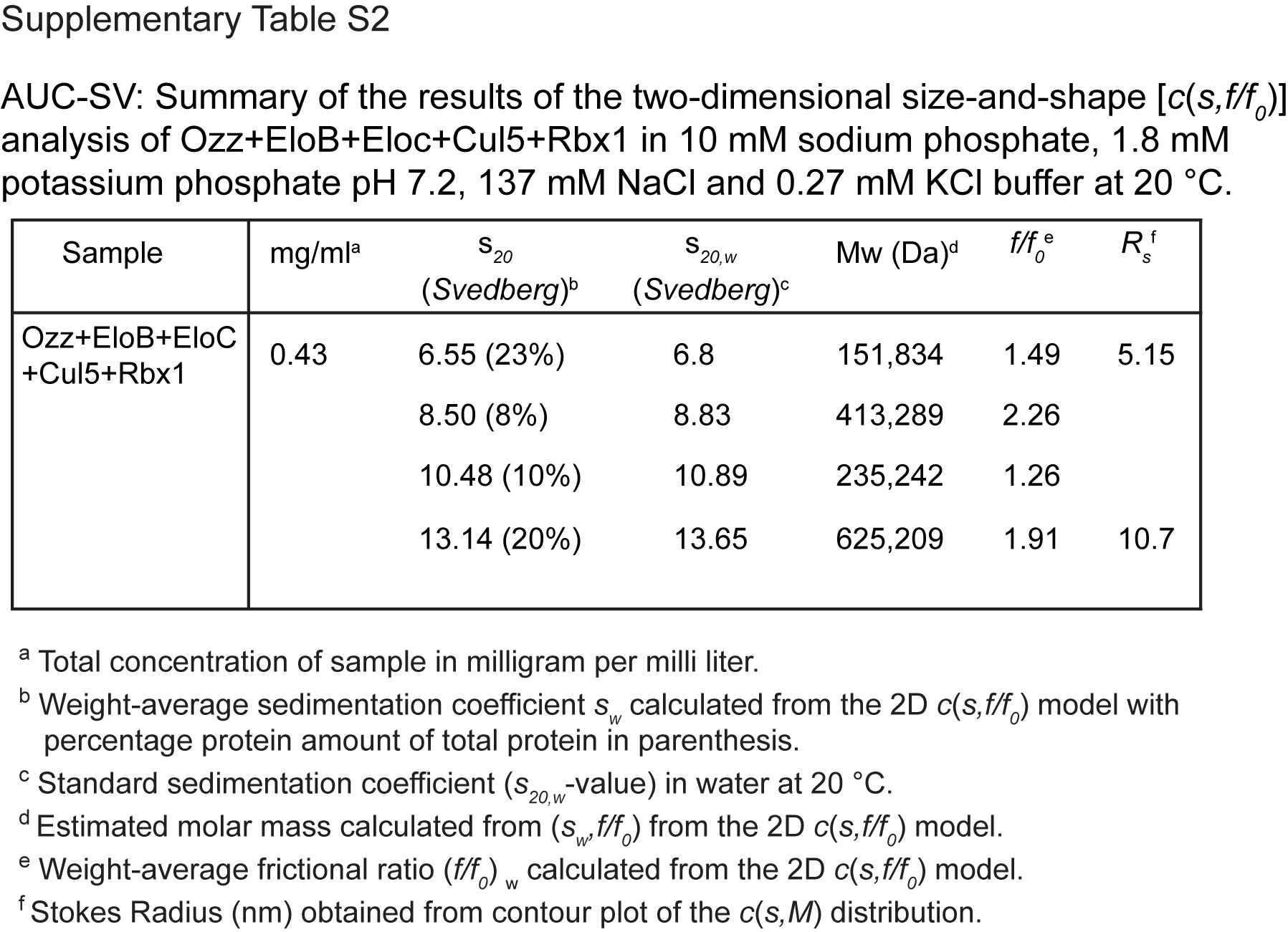

Supplement: Supplementary file 6 — Supplementary Table S2. [file 41598_2022_10955_MOESM6_ESM.tif]
