# Supplementary material for: Biophysical and functional study of CRL5Ozz, a muscle specific ubiquitin ligase complex
Source: Sci Rep. 2022 May 12;12:7820. doi: 10.1038/s41598-022-10955-w (PMC9098882; doi:10.1038/s41598-022-10955-w)
Supplement: Supplementary file 4 — Supplementary Information. [file 41598_2022_10955_MOESM4_ESM.pdf]

Figure 1

(a) Ozz-His tag

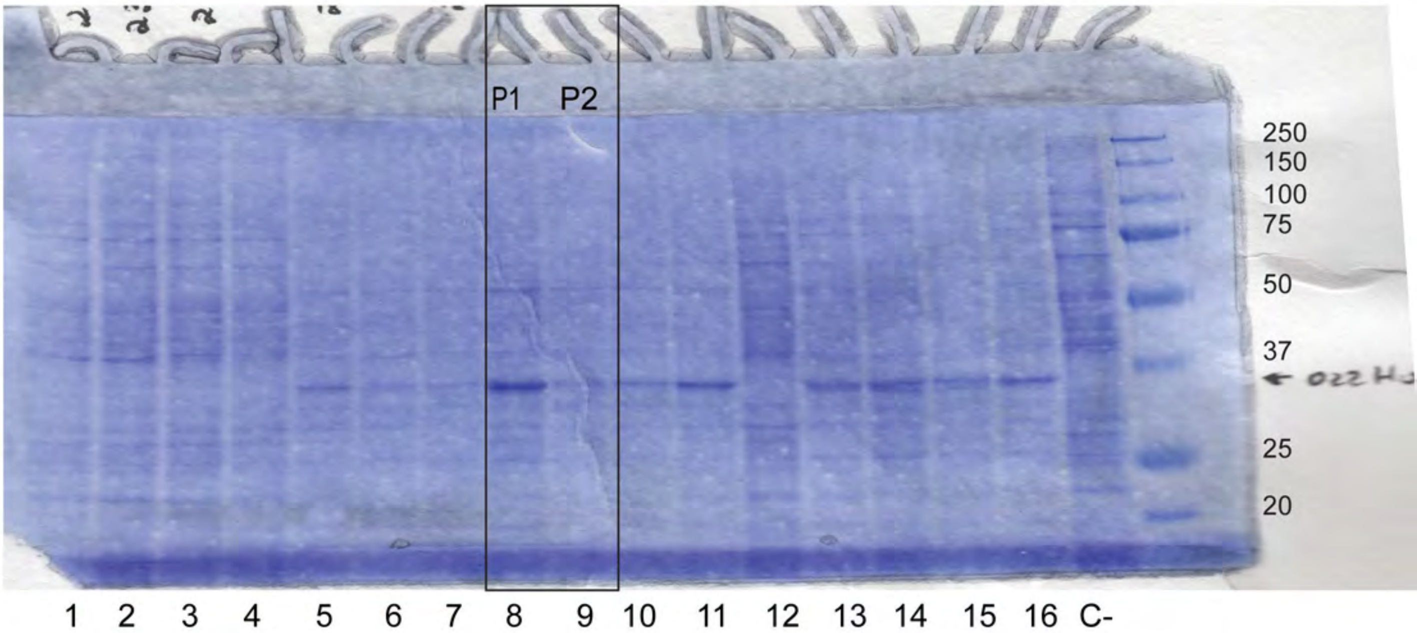

Coomassie brilliant blue staining: CBB

EloB

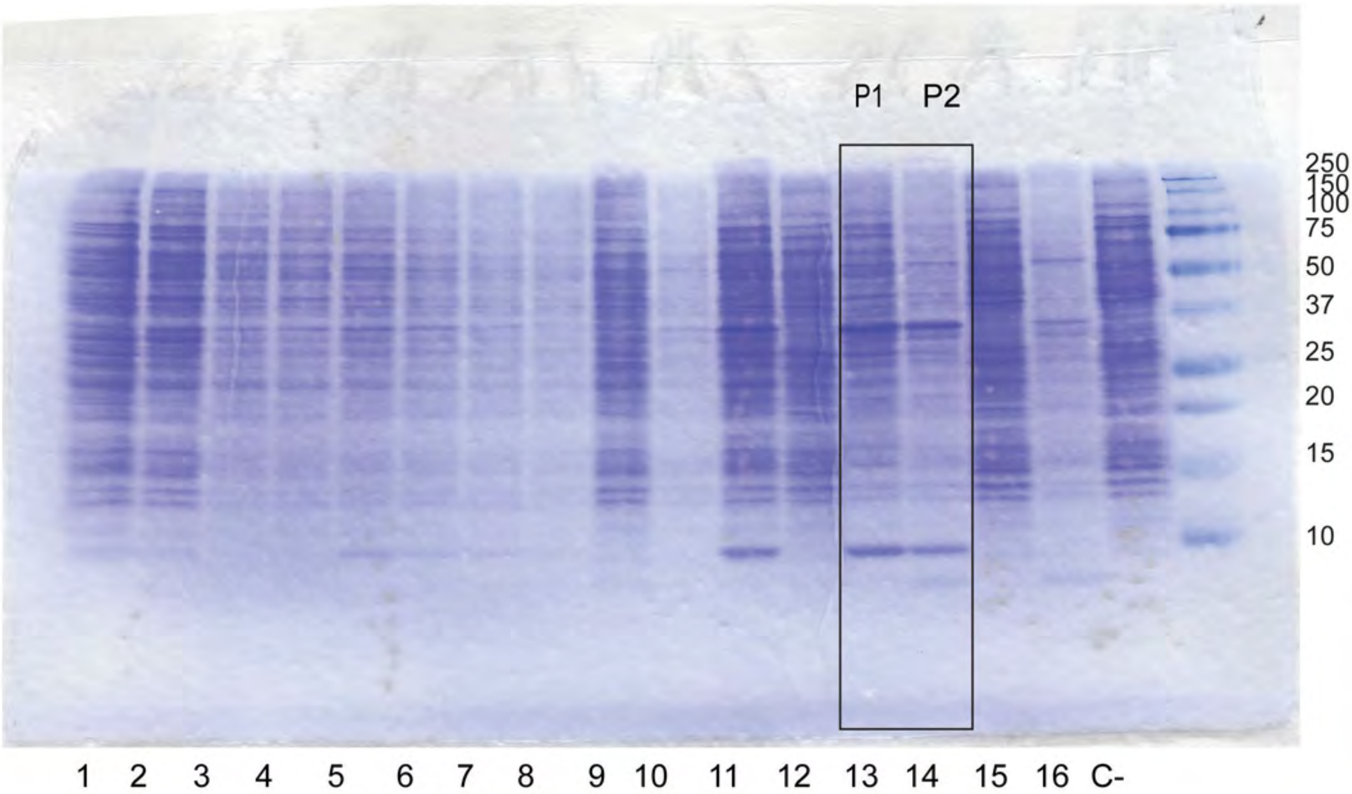

Coomassie brilliant blue staining: CBB

Uncropped original gels belonging to figure 1a.
The black outlines demarcate the selected area shown in figure 1a.

Figure 1

(a)

EloC

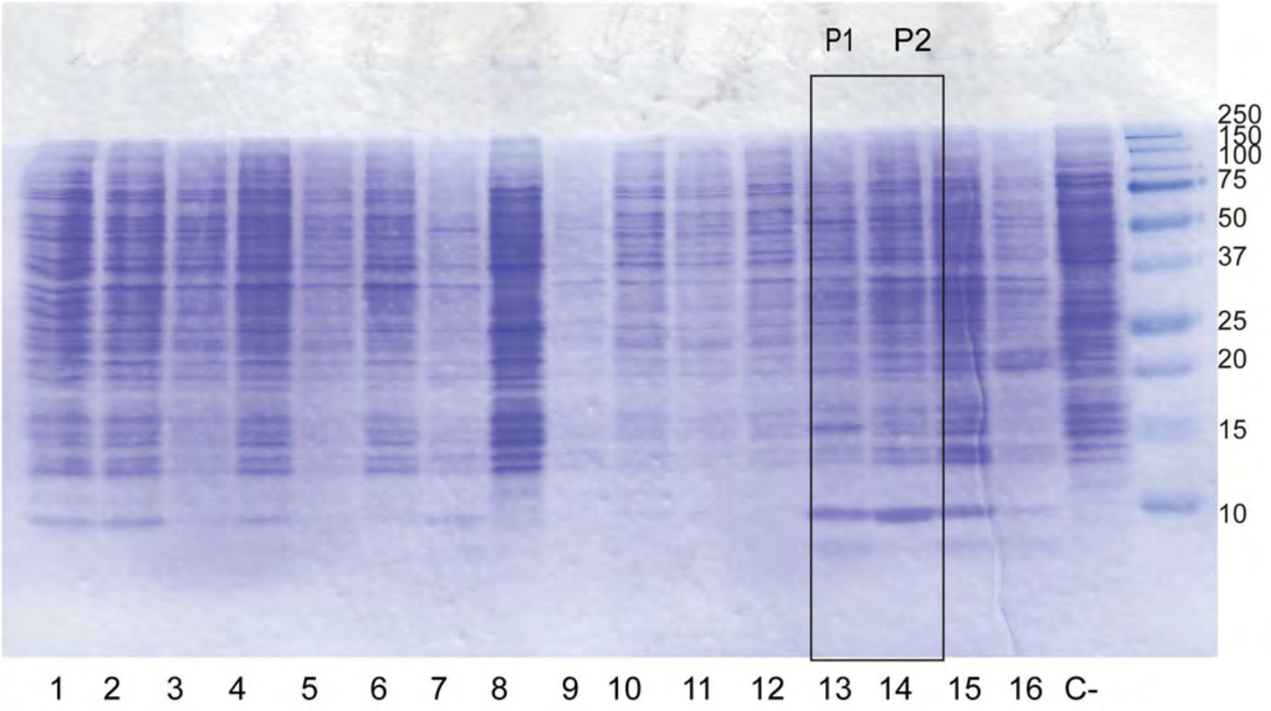

Coomassie brilliant blue staining: CBB

Cul5

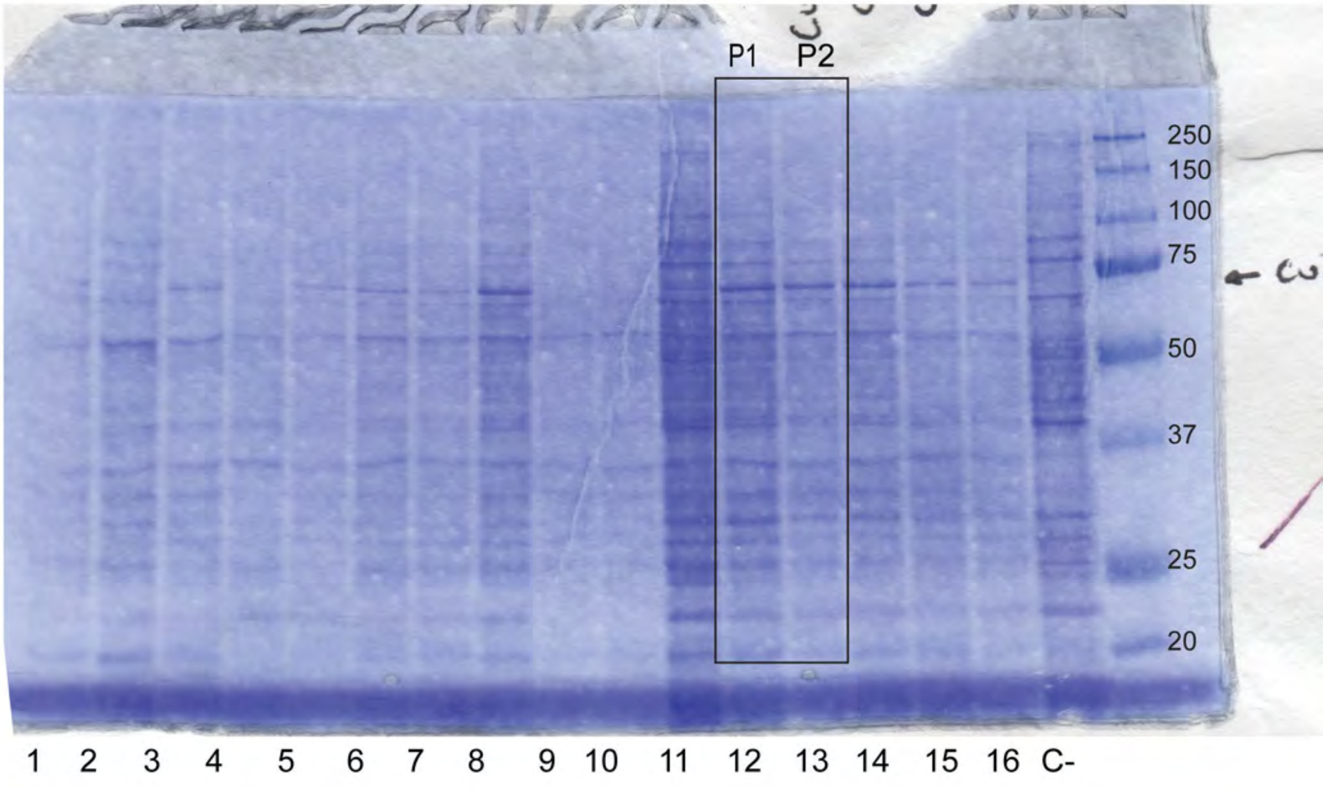

Coomassie brilliant blue staining: CBB

Uncropped original gels belonging to figure 1a.
The black outlines demarcate the selected area shown in figure 1a

Figure 1

(a) Rbx1

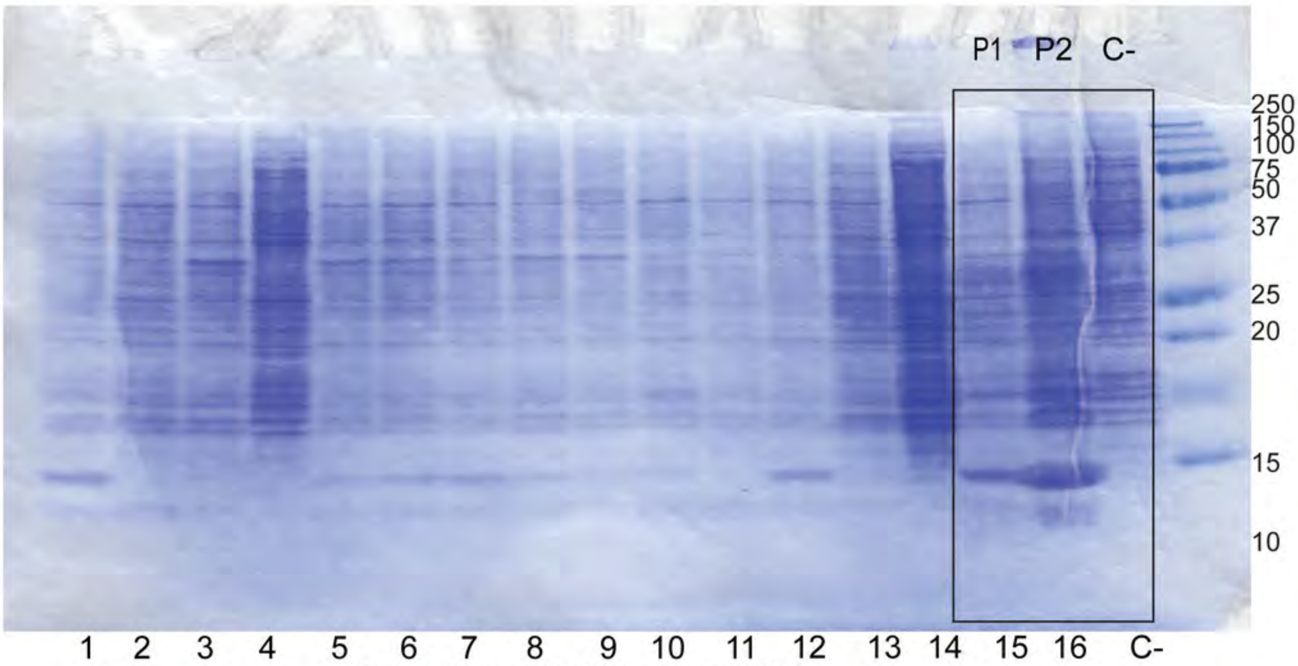

Coomassie brilliant blue staining: CBB

Uncropped original gel belonging to figure 1a. The black outline demarcates the selected area shown in figure 1a

(b)

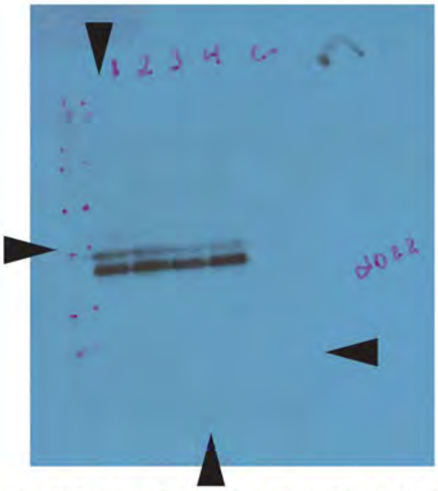

Black arrowheads mark the borders of the blot

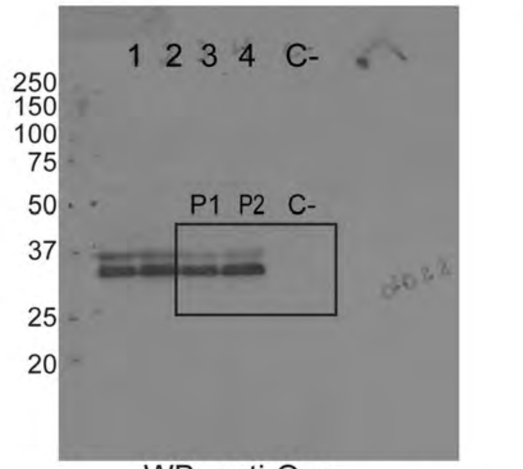

WB: anti-Ozz

Adjusted color to black&white and rotated with Adobe Photoshop 2022

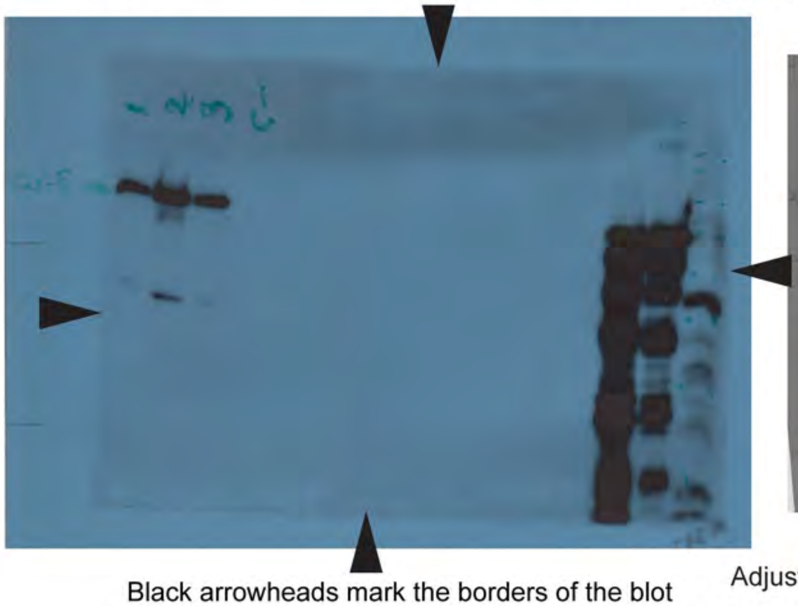

Black arrowheads mark the borders of the blot

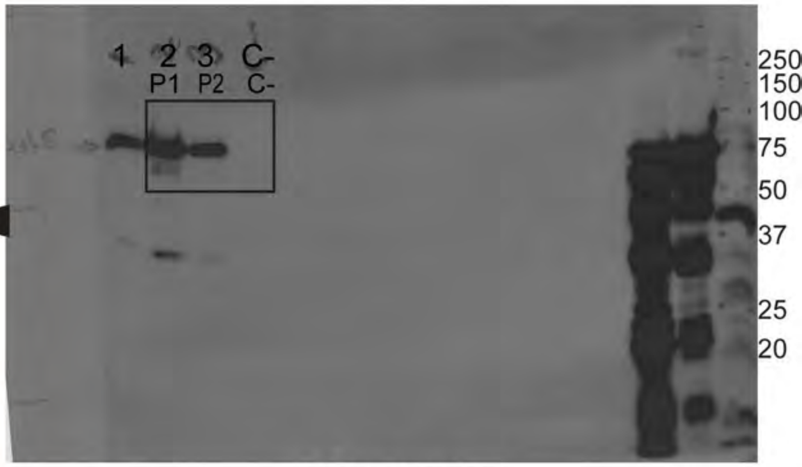

WB: anti-Cul5

Adjusted color to black&white and cropped with Adobe Photoshop 2022

Uncropped original Western Blot belonging to figure 1b.

Black outlines indicate the selected areas shown in figure 1b

Figure 1

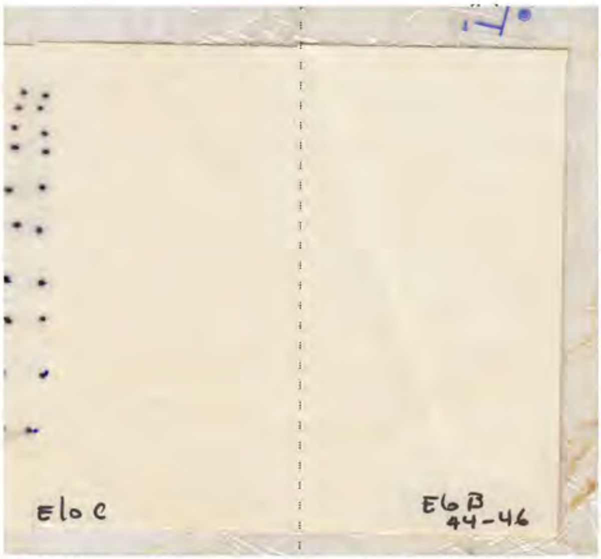

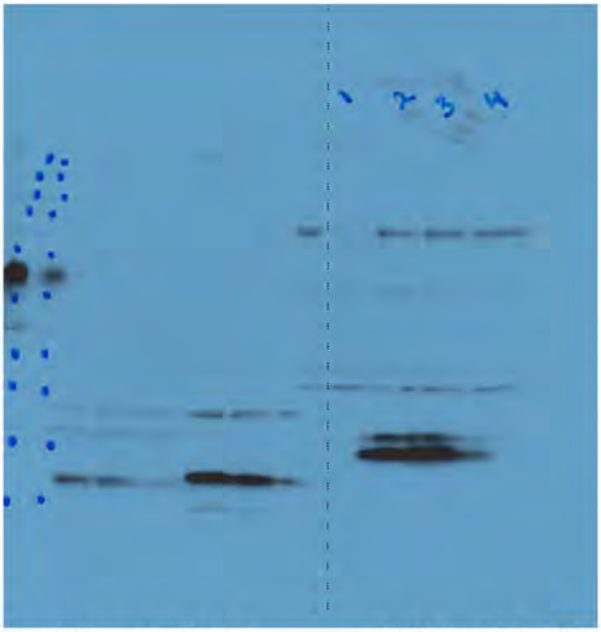

WB: anti-EloC WB: anti-EloB

PVDF membrane corresponding to the WB probed with anti-EloC or anti-EloB.
The dashed line indicates where the membrane was cut. The left half of the membrane was probed with anti-EloC and the rigth half was probed with anti-EloB.

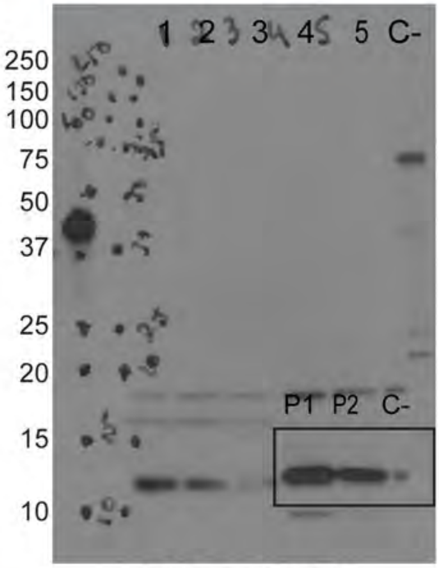

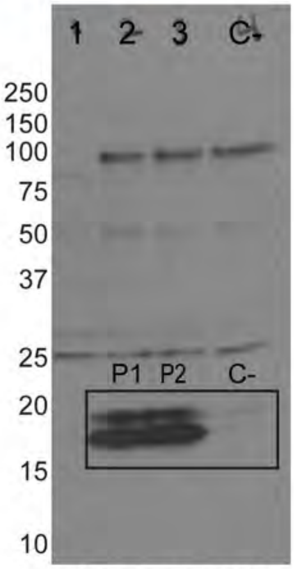

WB: anti-EloC WB: anti-EloB

Adjusted color to black&white and cropped with Adobe Photoshop 2022

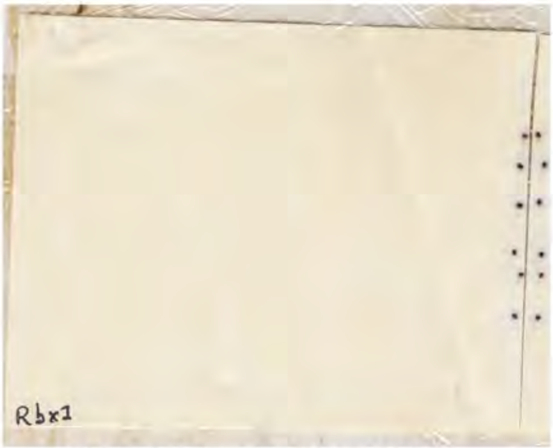

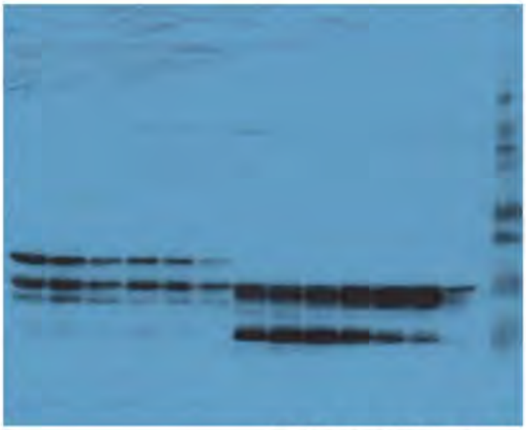

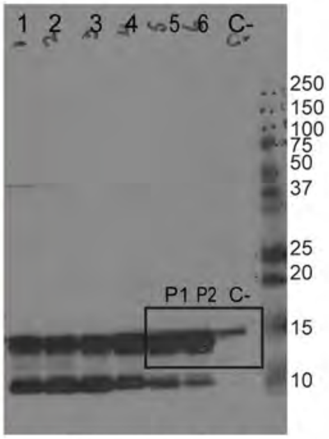

PVDF membrane corresponding to the WB probed with anti-Rbx1

WB: anti-Rbx1 WB: anti-Rbx1

Adjusted color to black&white and cropped with Adobe Photoshop 2022

Uncropped original Western Blots belonging to figure 1b.
The black outlines demarcate the selected areas shown in figure 1b.

Figure 1

(c)

Ozz-EloBC

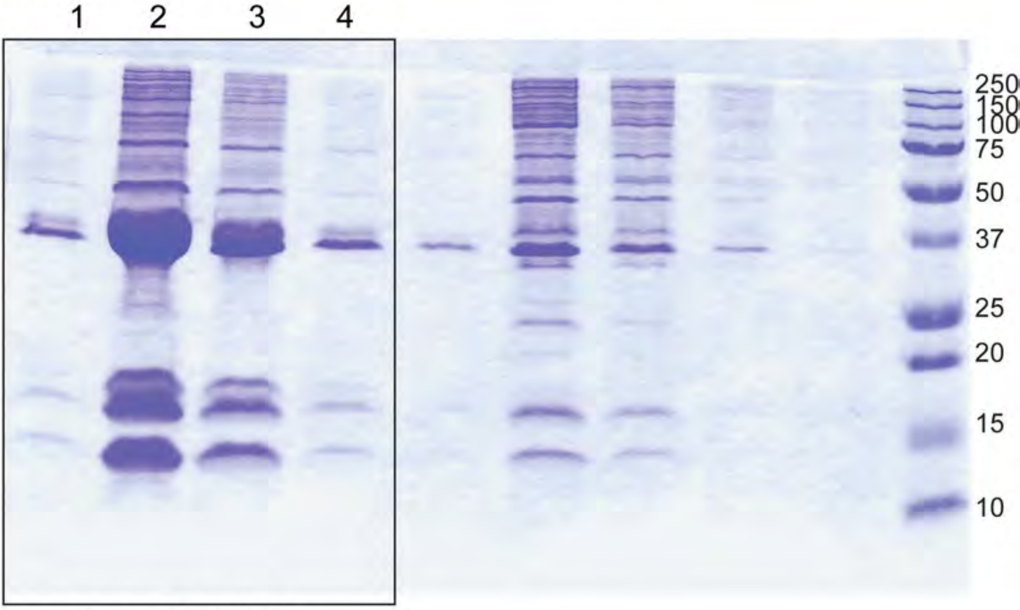

Eluted fractions

Coomassie brilliant blue staining: CBB

Uncropped original gel belonging to figure 1c.
The black outline demarcates the selected area shown in the figure 1c.

(d)

Cul5-Rbx1

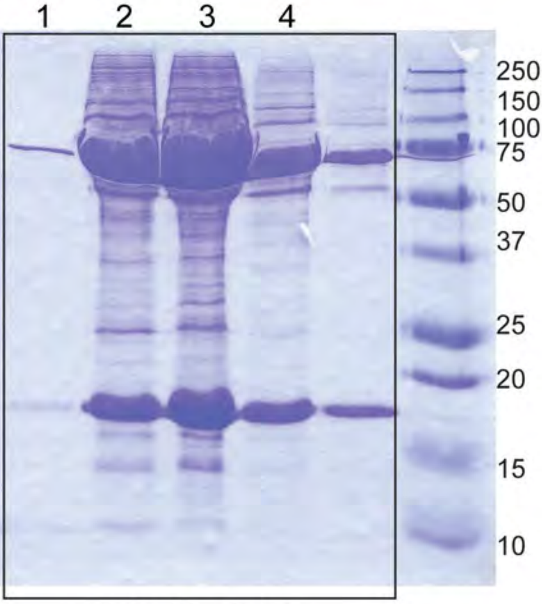

Eluted fractions

Coomassie brilliant blue staining: CBB

Uncropped original gel belonging to figure 1d.
The black outline demarcates the selected area shown in the figure 1d

Figure 2

(c)

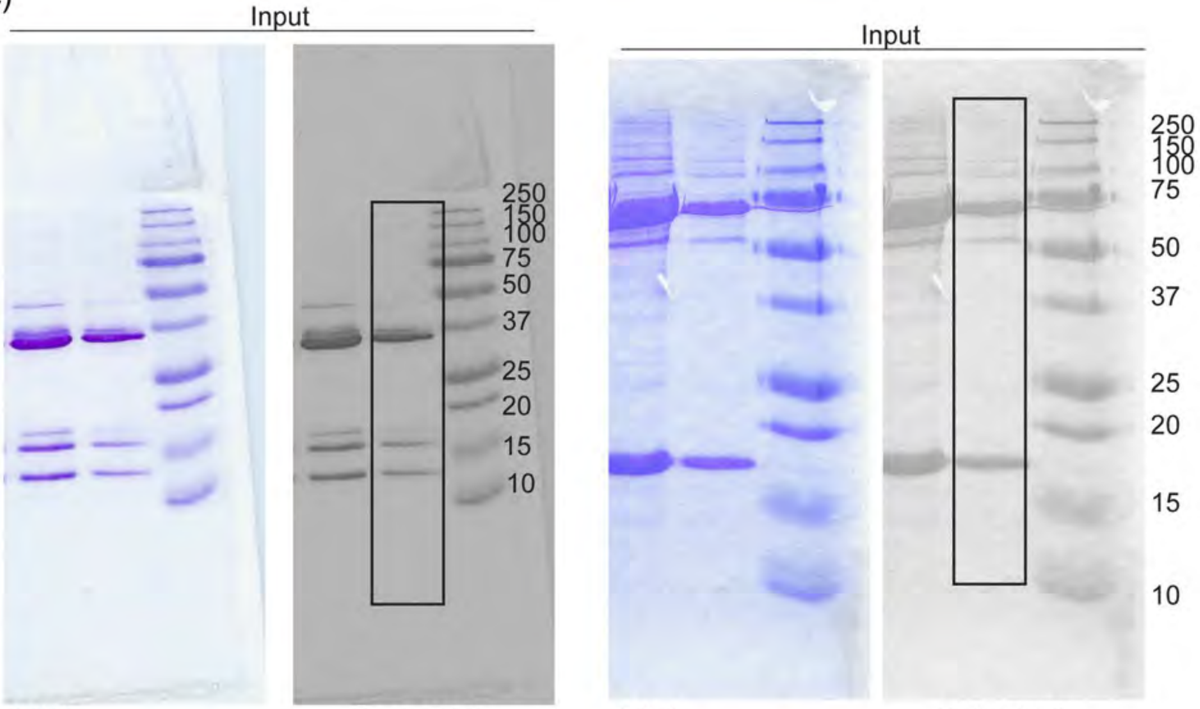

Change color to black and white in power point

Uncropped original gels belonging to figure 2c. The black outlines demarcate the selected areas shown figure 2c.

(d)

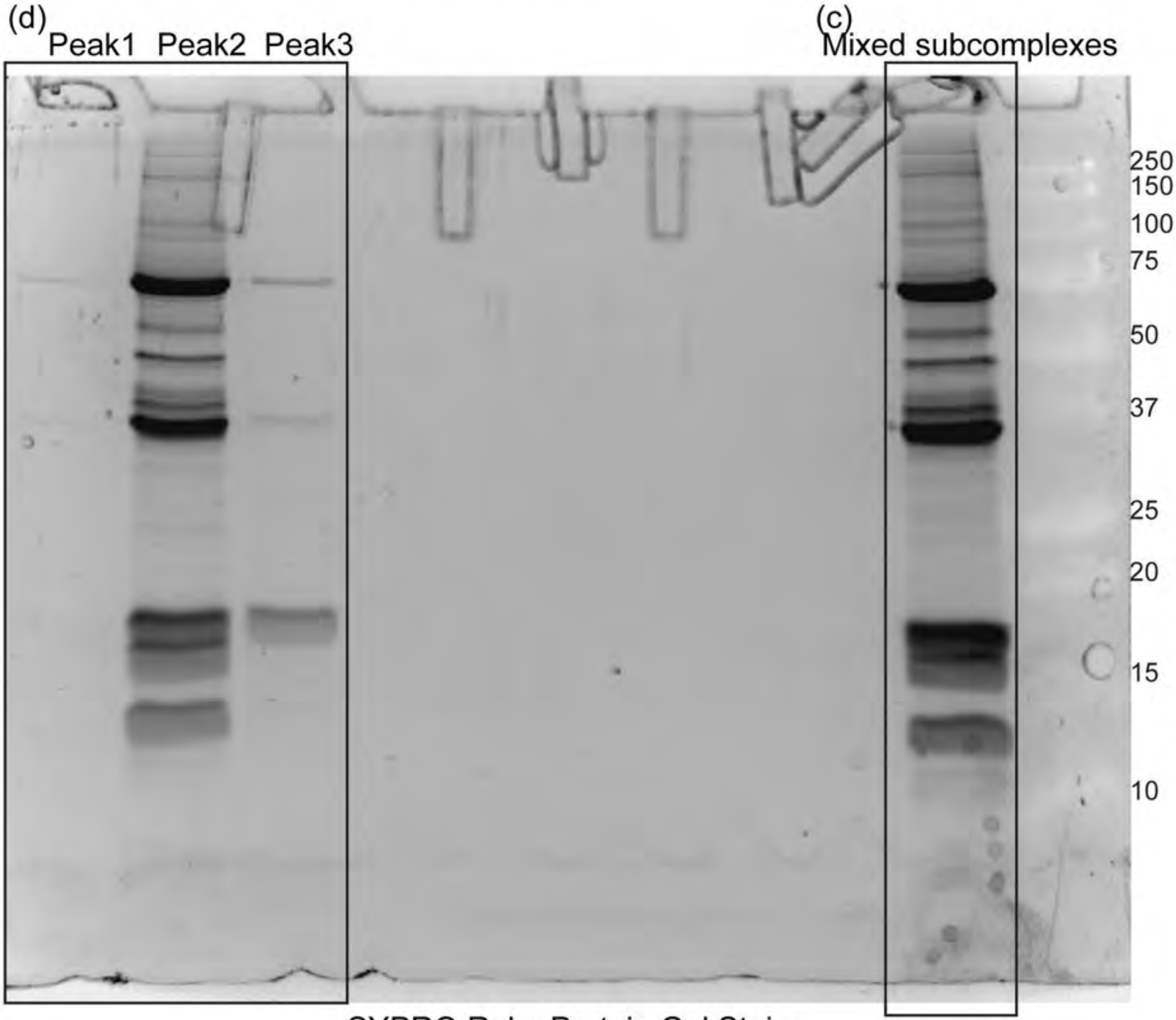

SYPRO Ruby Protein Gel Stain

Uncropped original gel belonging to figure 2c,d.
The black outlines demarcate the selected areas shown figure 2c,d

Figure 2

(e)

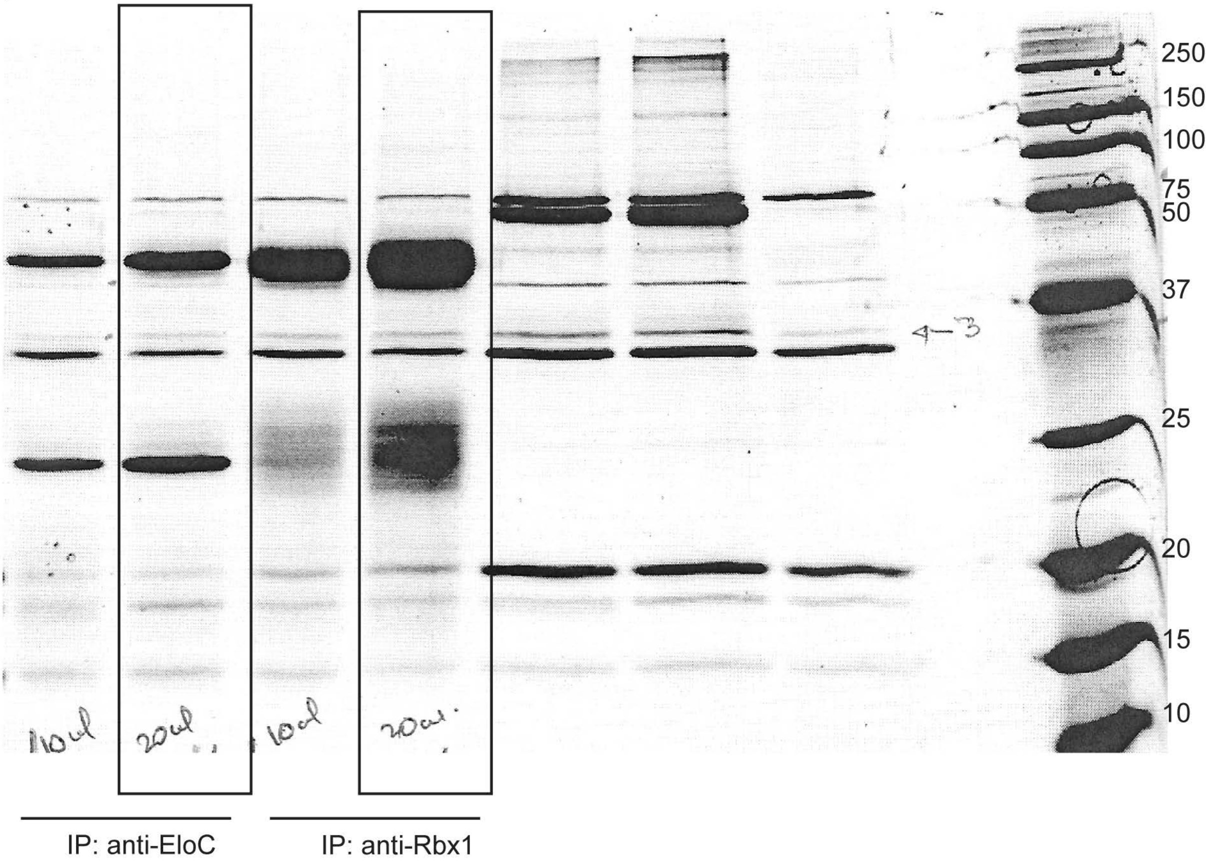

SYPRO Ruby Protein Gel Stain

Uncropped original gel belonging to figure 2e.
The black outlines demarcate the selected areas shown in figure 2e

Figure 3

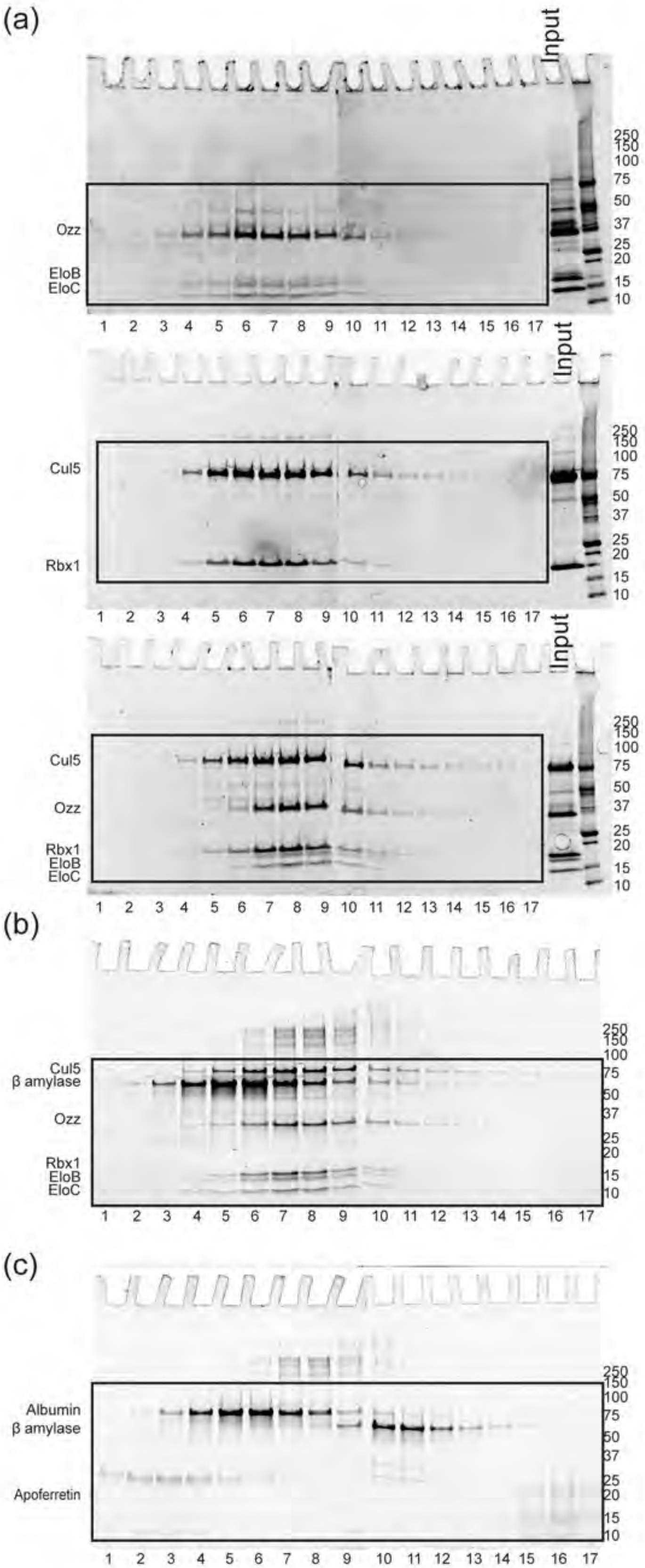

SYPRO Ruby Protein Gel Stain

Uncropped original gels belonging to figure. 3a-c.
The black outlines demarcate the selected areas shown in the figure.

Figure 6

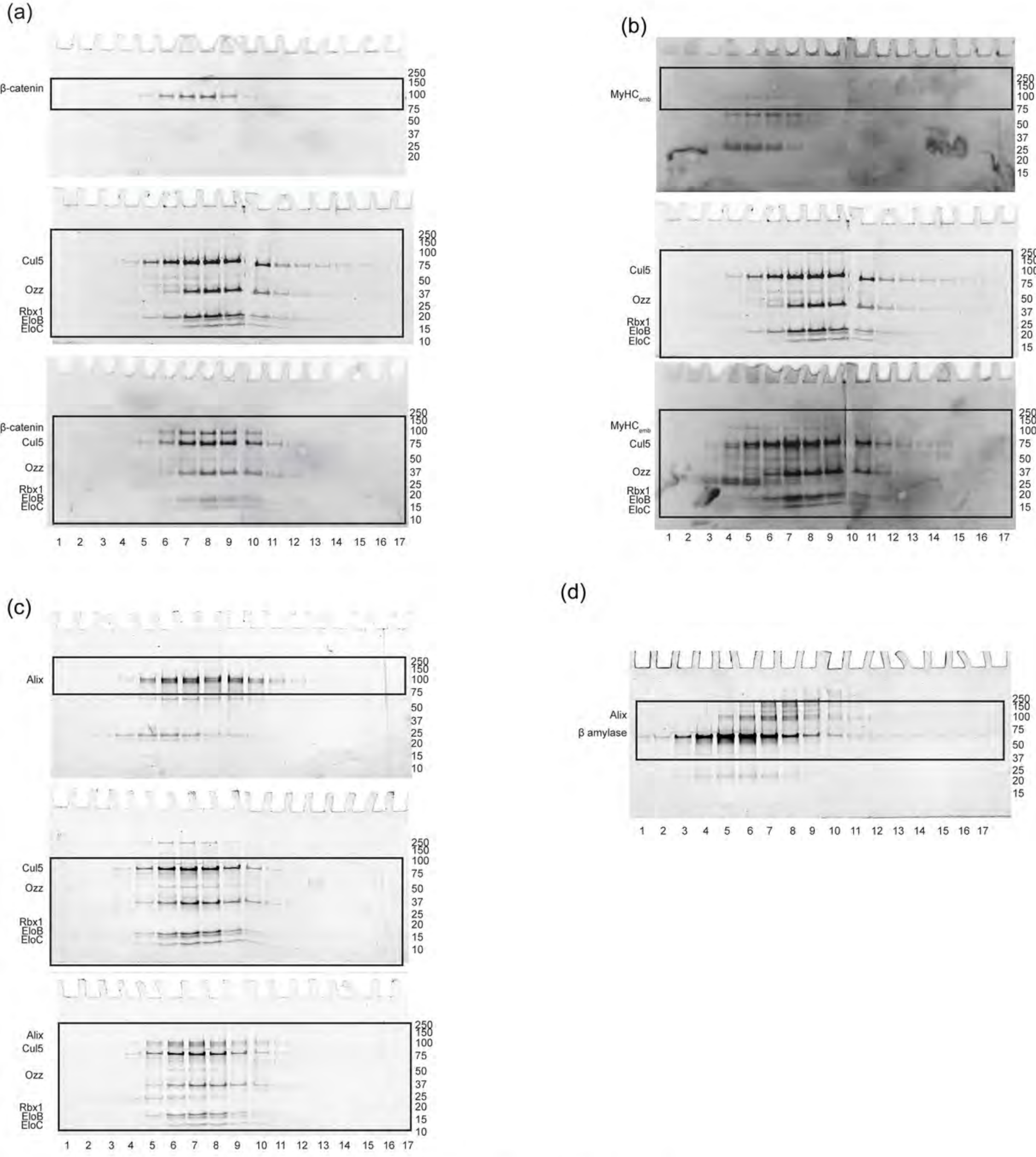

SYPRO Ruby Protein Gel Stain

Uncropped original gels belonging to figure 6a-d.
The black outlines demarcate the selected areas shown in figure 6a-d.

Figure 7

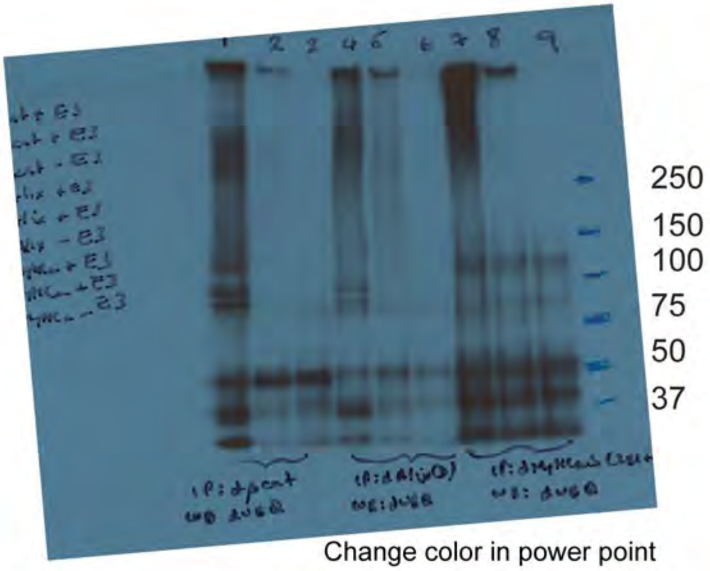

Change color in power point

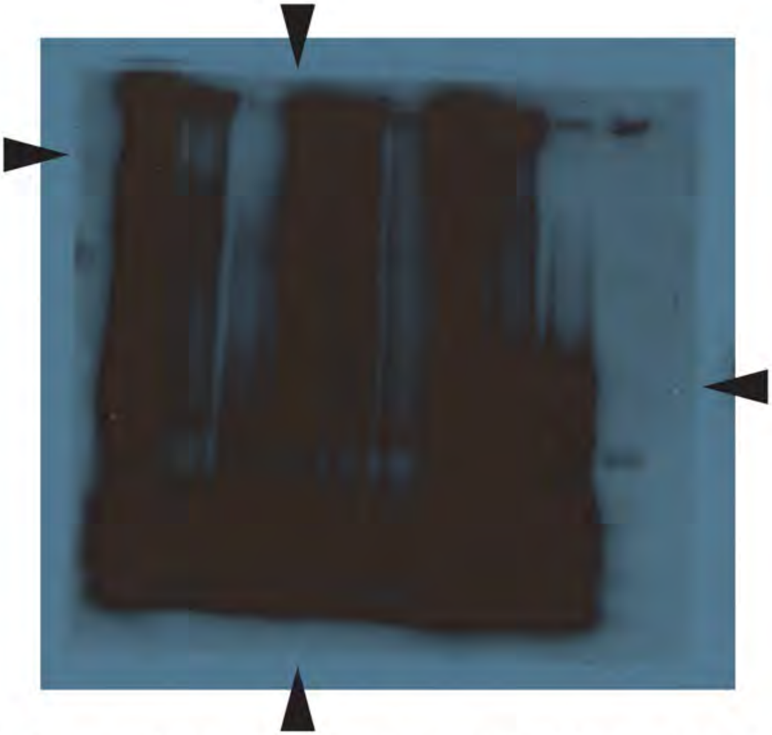

Overexposed Western Blot showing the borders (black arrowheads)

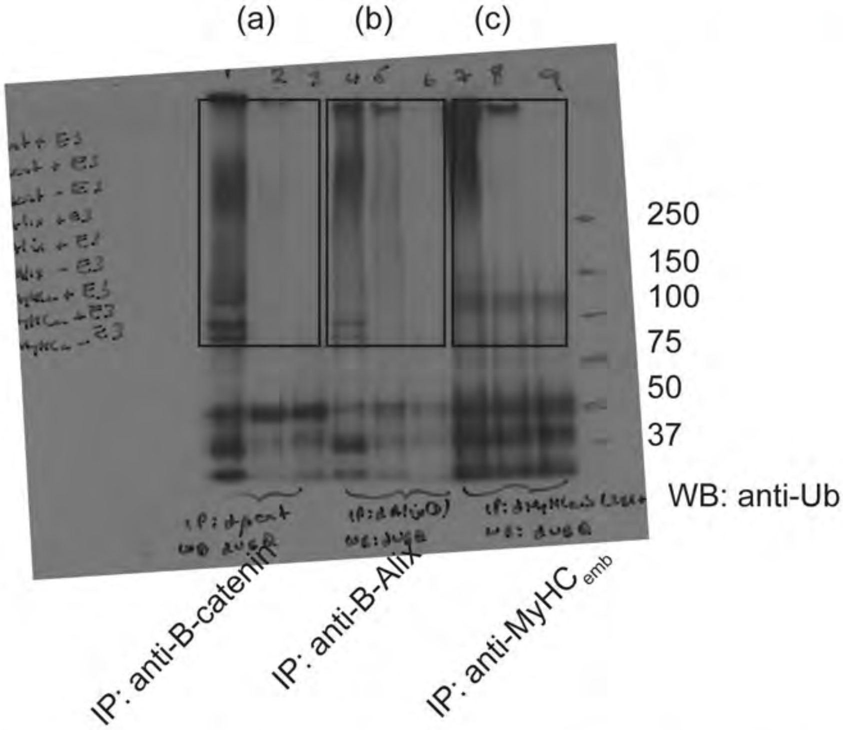

Adjusted color to black&white and roateded with Adobe Photoshop 2022

Uncropped original western blot film belonging to figure 7a-c.
The black outlines demarcate the selected areas shown in figure 7a-c.

Figure 7

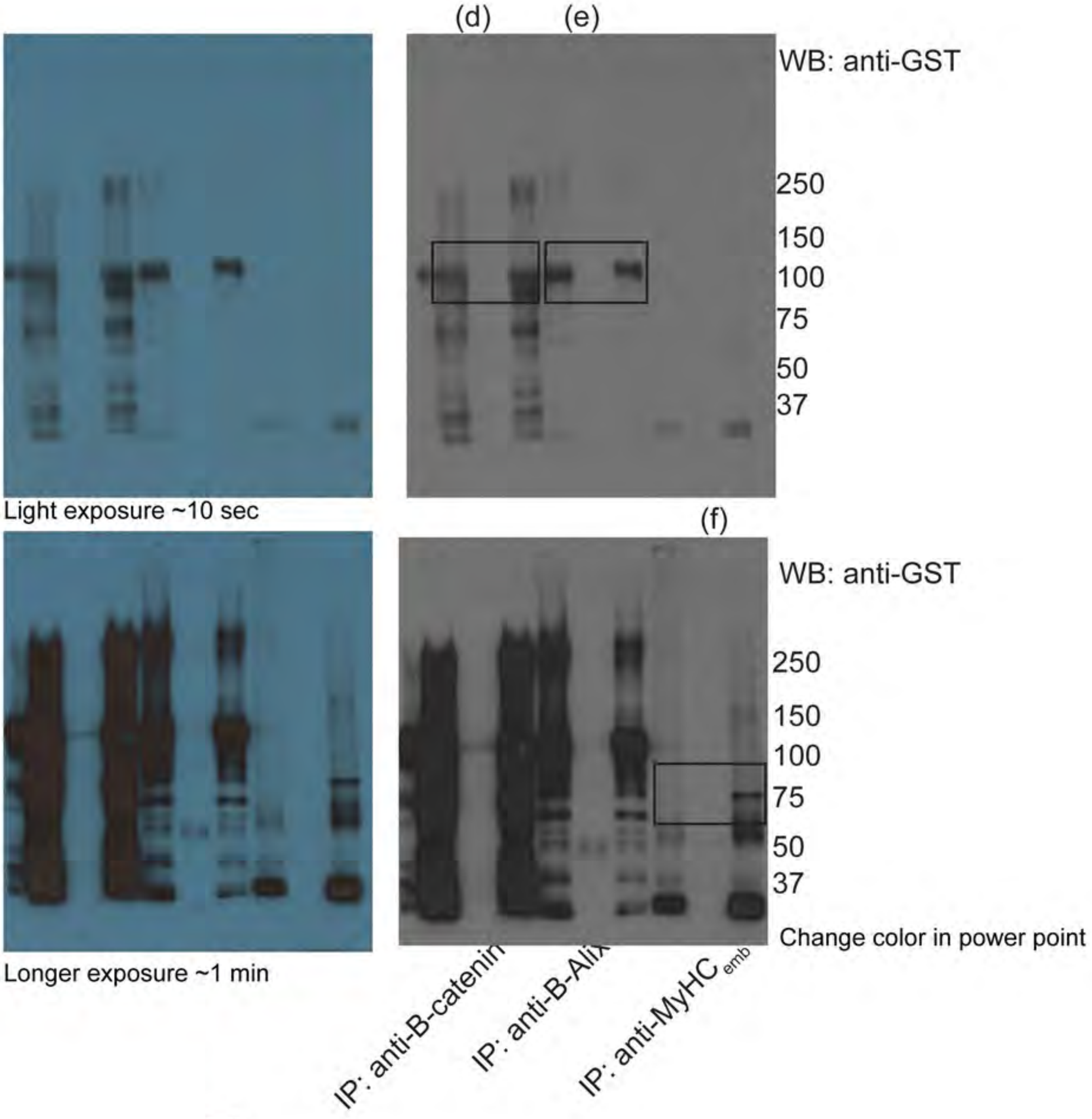

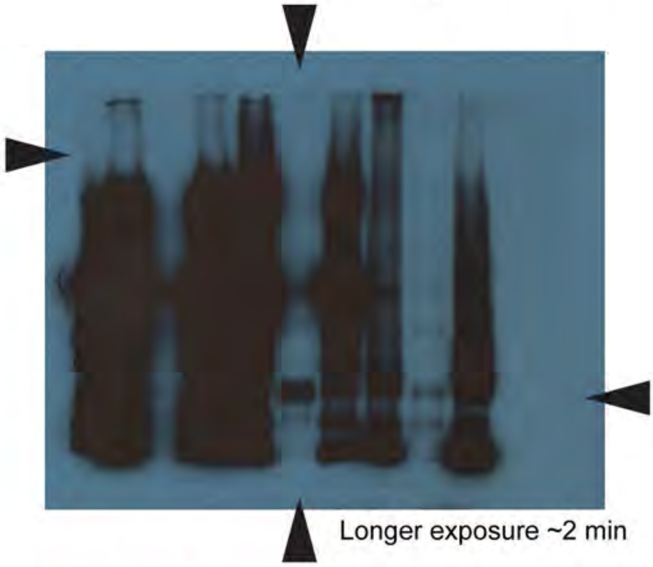

Overexposed Western Blot showing the borders (black arrowheads)

Uncropped original western blot film belonging to figure 7d-f.
The black outlines demarcate the selected areas shown in figure 7d-f.

Figure 7
(a)

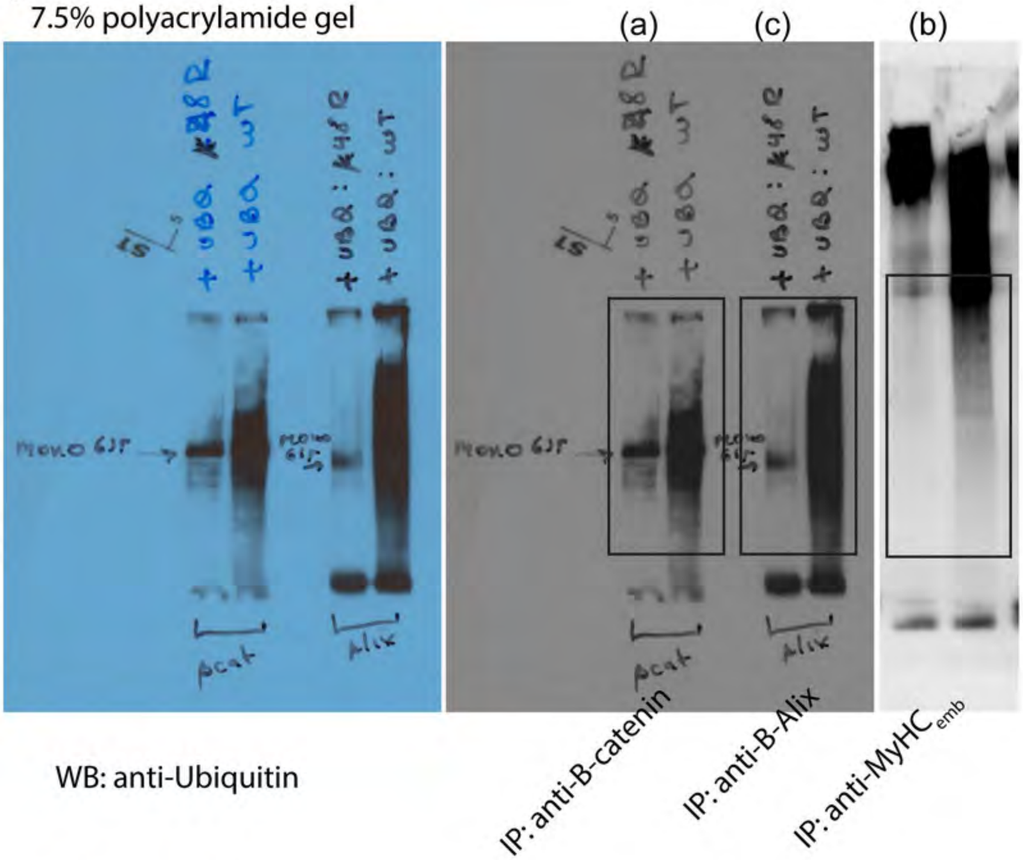

Change color to black and white in power point.
The black outlines demarcate the selected areas shown in figure 7a-c.

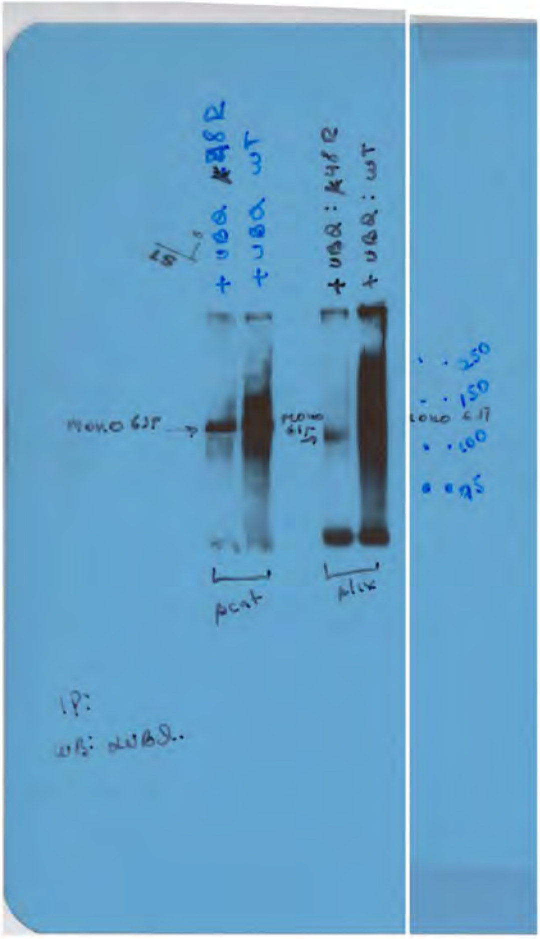

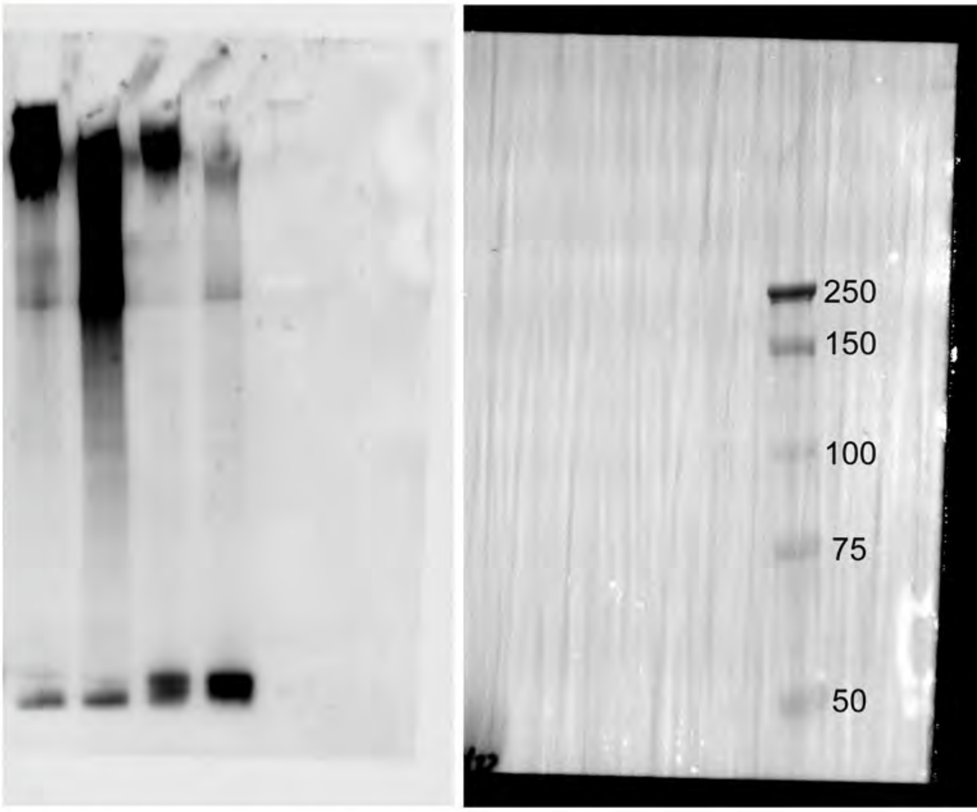

Uncropped original film corresponding to the Western Blot of the mono UBQ of Ozz-E3 subtrates

Uncropped original Western Blot belonging to figure 7a-c.

Figure 7

(d) (f) (e)

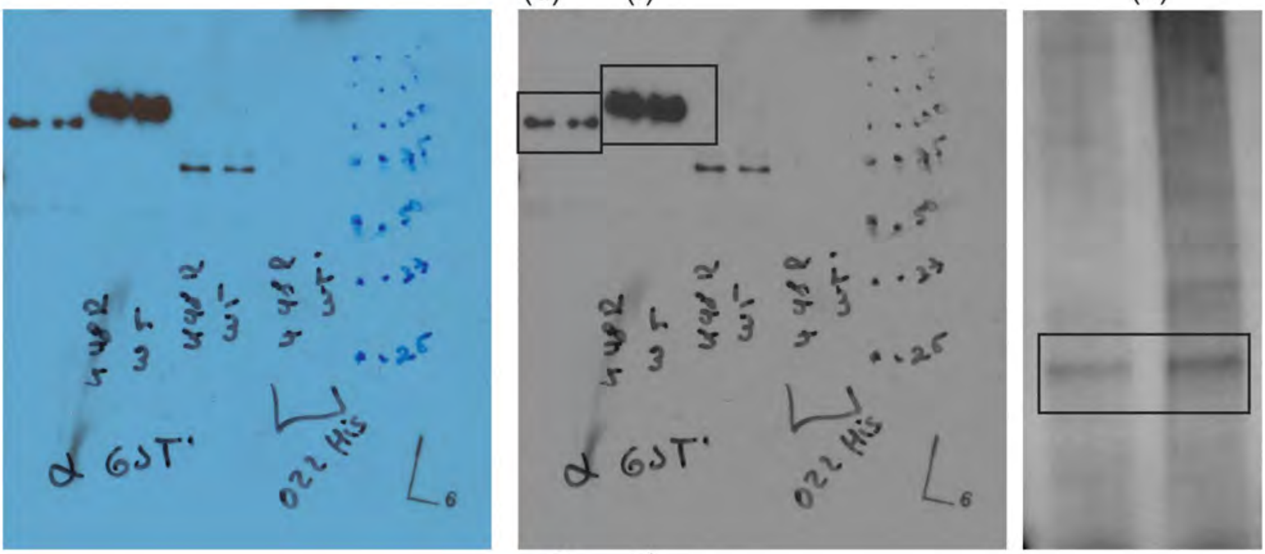

WB: anti-GST

IP: anti-B-catenin

IP: anti-B-Alix

IP: anti-MyHC$_{emb}$

Change color to black and white in power point.
The black outlines demarcate the selected areas shown in figure 7d-f.

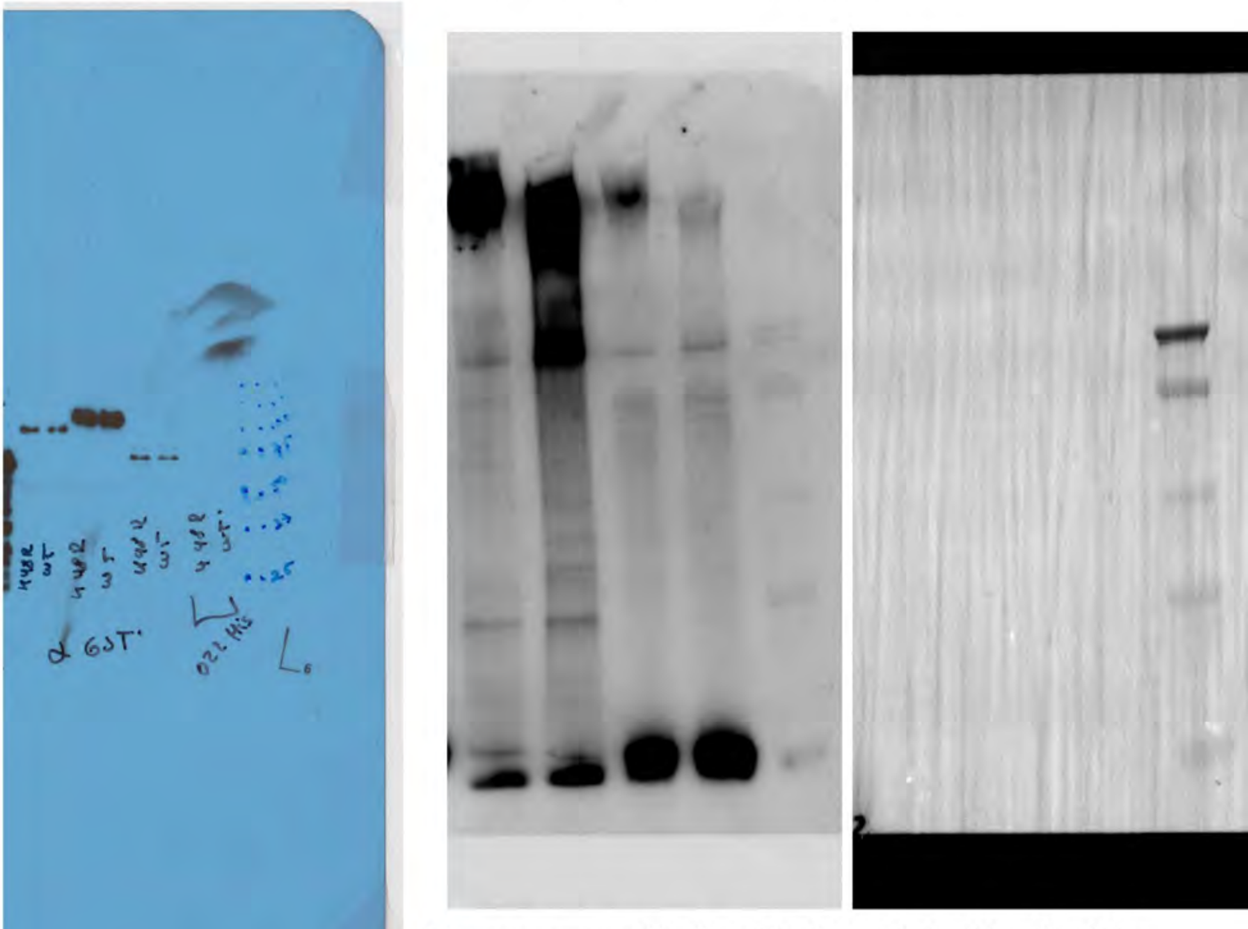

Film correspond to the Western Blot of the IP of the mono UBQ of Ozz-E3 subtrates

Uncropped original Western Blot belonging to Fig. 7d-f.
